# Supplementary material for: Flavonoid-Rich Extract from Bombyx batryticatus Alleviate LPS-Induced Acute Lung Injury via the PI3K/MAPK/NF-κB Pathway
Source: Int J Mol Sci. 2025 Dec 15;26(24):12057. doi: 10.3390/ijms262412057 (PMC12732911; doi:10.3390/ijms262412057)
Supplement: Supplementary file 1 [file ijms-26-12057-s001.zip › ijms-3973997-supplementary.pdf]

Flavonoids-rich extract from *Bombyx batryticatus* alleviate LPS-induced acute lung injury via the PI3K/MAPK/NF-κB pathway

Supplementary

Contents

Supplementary Figure 2
Figure S1 2
Figure S2 3
Figure S3 4
Supplementary Table 5
Table S1 5
Table S2 10
Table S3 45
Table S4 46
Table S5 47
The ARRIVE guidelines 2.0: author checklist 48

Supplementary Figure

Figure S1

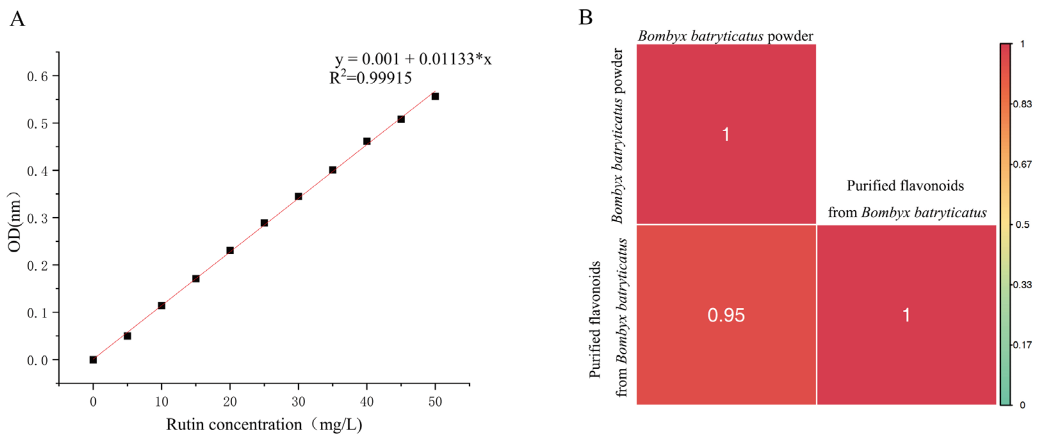

Figure S1. Quantification of total FBB. (A) Rutin standard curve. (B) Correlation between crude and purified FBB extracts.

Figure S2

A

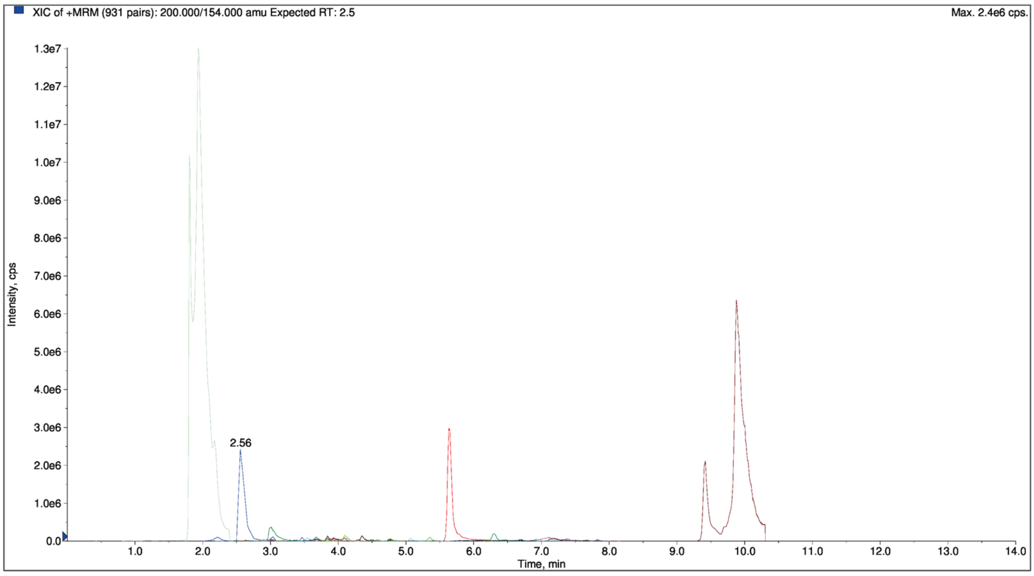

B

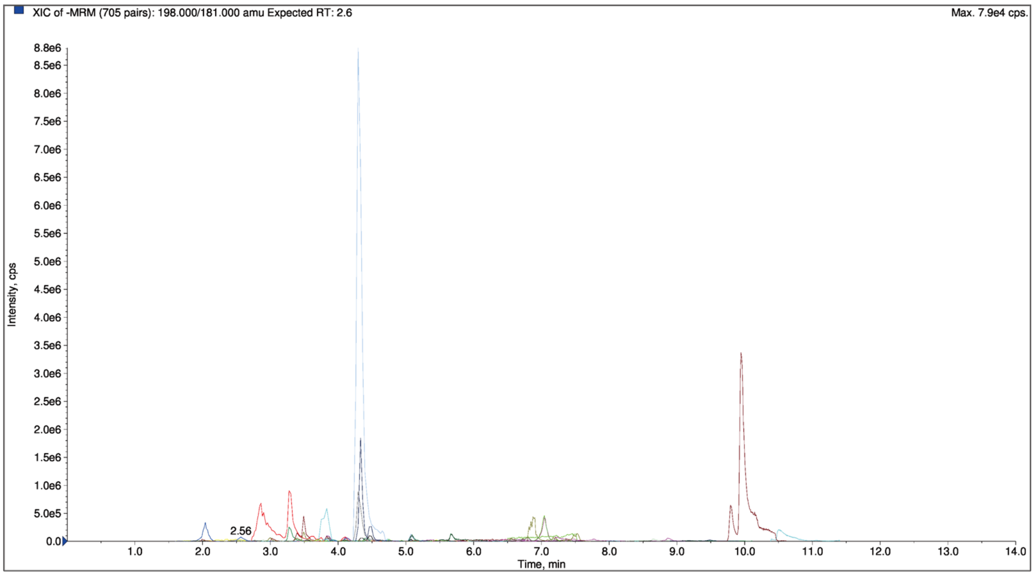

Figure S2 .MRM metabolite detection multi-peak diagram .(A) is positive ion mode. (B) is negative ion mode.

Figure S3

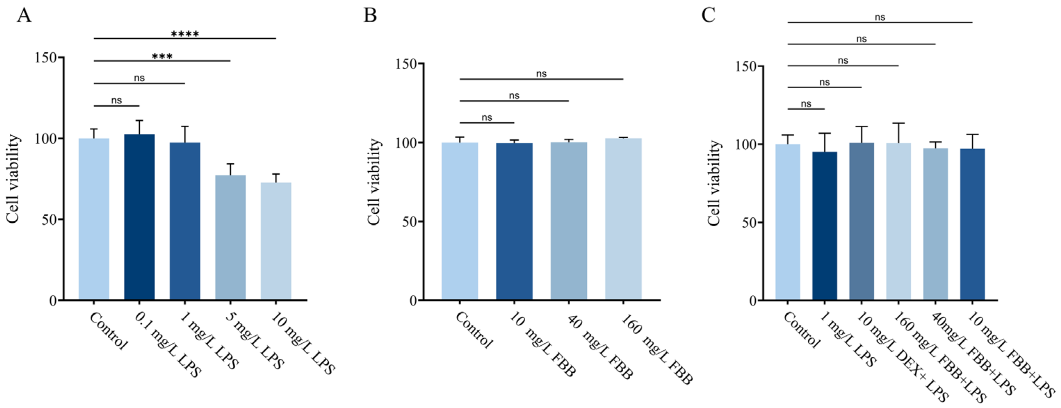

Figure S3. Assessment of cell viability in RAW264.7 macrophages treated with FBB and LPS. (A) LPS concentration screening experiment. (B) Cytotoxicity assessment of FBB in RAW264.7 cells. (C) Effects of FBB and LPS co-treatment on cell viability.

Supplementary Table

Table S1

Qualitative analysis of FBB

ID	Compounds	Class II	Relative content （Area）
FBB1	(2S)-Abyssinone II	Flavanones	179459.6
FBB2	(3S)-5,7-dihydroxy-3-[(4-methoxyphenyl)methyl]-6-methyl-2,3-dihydrochromen-4-one*	Other Flavonoids	235328.1
FBB3	1,2,3,7,8-pentahydroxy-6-methylanthracene-9,10-dione*	Other Flavonoids	105495.8
FBB4	1,2,4,5,8-pentahydroxy-6-methylanthracene-9,10-dione*	Other Flavonoids	1094816
FBB5	1,2,4,5-tetrahydroxy-7-(hydroxymethyl)anthracene-9,10-dione*	Other Flavonoids	23799.53
FBB6	1,2,5,7,8-pentahydroxy-3-methylanthracene-9,10-dione	Other Flavonoids	915050.8
FBB7	1-hydroxy-7-{[(2s,3r,4s,5r,6r)-3,4,5-trihydroxy-6-(hydroxymethyl)oxan-2-yl]oxy}xanthen-9-one	Other Flavonoids	3682.516
FBB8	2',3',4',5,7-Pentahydroxyflavone*	Flavones	947005.4
FBB9	2,4,2',4'-tetrahydroxy-3'-prenylchalcone	Chalcones	308357.3
FBB10	2',7-Dihydroxy-3',4'-dimethoxyisoflavan	Other Flavonoids	20359.17
FBB11	2'-Hydroxy-3,4,5,3',4',6'-hexameth-oxychalcone	Chalcones	46774.24
FBB12	Polygonatone C	Other Flavonoids	47333.06
FBB13	Ranupetin	Flavonols	35843.69
FBB14	3,5-Diacetyltambulin	Flavones	169990.1
FBB15	3'-Hydroxy-4'-O-methylglabridin	Other Flavonoids	19964.05
FBB16	3-Methylkaempferol	Flavonols	7769.736
FBB17	3-O-Acetylpinobanksin	Flavanonols	224450.4
FBB18	3'-O-Methyldiplacol	Flavanonols	223183.6
FBB19	3-O-Methylquercetin	Flavonols	83976.69
FBB20	4'-Hydroxy-5,7-dimethoxyflavanone	Flavanones	15982.14
FBB21	5,6,7,5'-tetramethoxy-3',4'-methylenedioxyflavonoid	Flavones	31180.25
FBB22	5,7,3',4'-Tetrahydroxy-6-methoxyflavone-8-C-[Xylosyl-(1-2)]-glucoside	Flavones	386276.4
FBB23	5,7,5'-Trimethoxy-3',4'-methylenedioxyflavonoid	Flavones	22421.07
FBB24	5,7-dihydroxy-3-(4'-hydroxybenzyl)-chroman-4-one	Other Flavonoids	20005

ID	Compounds	Class II	Relative content （Area）
FBB25	5,7-Dihydroxy-6,8-dimethoxyflavone-7-O-glucoside	Flavones	18840.43
FBB26	5-hydroxy-2-methoxyxanthen-9-one	Other Flavonoids	61555.31
FBB27	5-hydroxy-7,9,10-trimethoxy-2,2-dimethyl-1,11-dioxatetracen-6-one	Other Flavonoids	2235034
FBB28	6,8-Dihydroxy-2-(2-hydroxy-4-methoxybenzyl)-7-methyl-3,4-dihydronaphthalen-1(2H)-one	Other Flavonoids	42165.89
FBB29	6-C-Glucosyl-2-Hydroxynaringenin	Flavanones	2570129
FBB30	Mimulone	Flavanones	178800.9
FBB31	6-Hydroxykaempferol-3,6-O-Diglucoside*	Flavonols	1963019
FBB32	6-Hydroxykaempferol-3-O-Rutinoside-6-O-glucoside*	Flavonols	706411.9
FBB33	6-Hydroxykaempferol-6,7-O-Diglucoside*	Flavonols	90103.6
FBB34	6-Hydroxykaempferol-7-O-glucoside*	Flavonols	8384780
FBB35	6-Hydroxyluteolin 5-glucoside*	Flavones	2414514
FBB36	7,4'-Dihydroxydihydroflavonoids	Flavanones	14285.3
FBB37	7-Hydroxyflavone	Flavones	53545.18
FBB38	7-Methoxy-3-[1-(3-pyridyl)methylidene]-4-chromanone	Other Flavonoids	1.17E+08
FBB39	Apigenin-4'-O-(2'',6''-di-O-p-coumaroyl)glucoside	Flavones	9909.216
FBB40	Apigenin-7-O-(2''-Sinapoyl)glucuronide	Flavones	2890.961
FBB41	Aromadendrin	Flavanonols	9630.918
FBB42	Artemetin	Flavones	113260.4
FBB43	Avicularin	Flavonols	65537.83
FBB44	Bracteatin*	Other Flavonoids	105495.8
FBB45	Carthamone*	Chalcones	782426.1
FBB46	C-glucosyl-C-arabinosyl-2-hydroxynaringenin	Flavanones	2380.964
FBB47	Chrysin	Flavones	50754.16
FBB48	Chrysoeriol glucosyl xylosyl glucoside	Flavones	87008.84
FBB49	Chrysoeriol-7-O-glucoside	Flavones	281420.4
FBB50	Chrysoeriol-7-O-glucuronide*	Flavones	72526.27
FBB51	Clitorin*	Flavones	1147559
FBB52	Dihydrokaempferide	Flavanonols	57937.05
FBB53	Diosmetin-7-O-glucuronide*	Flavones	110920.3
FBB54	Disporopsin	Flavones	418451.9
FBB55	Eriodictyol-7-O-glucoside*	Flavanones	212748
FBB56	Eriodictyol-8-C-glucoside-4'-O-glucoside	Flavanones	24217.61
FBB57	Eucalyptin	Flavanones	121362.1
FBB58	Eupatorin-5-methylether	Flavones	1925.924

ID	Compounds	Class II	Relative content （Area）
FBB59	Gallocatechin-(4α→8)-gallocatechin	Flavanols	327462.4
FBB60	Glabrescione B	Isoflavones	2840610
FBB61	Glucosyl-(2R)-Phellodensin-F	Flavanones	94717.56
FBB62	Gossypetin(3,3',4',5,7,8-Hexahydroxyflavone)	Flavonols	49993.97
FBB63	Gossypetin-8-O-β-D-glucuronide	Flavonols	80301.86
FBB64	Hesperetin-5-O-glucoside	Flavanones	2196126
FBB65	Hesperetin-7-O-glucoside	Flavanones	316161.2
FBB66	Isobavachalcone	Chalcones	32003.97
FBB67	Isobavachalcone glucoside	Chalcones	81038.16
FBB68	Isobavachin	Flavanones	46564.36
FBB69	Isohyperoside*	Flavonols	7756963
FBB70	iso-ophiopogonanone B*	Other Flavonoids	174515.5
FBB71	Isoorientin-7-O-glucoside*	Flavones	1311838
FBB72	Isorhamnetin-3-O-(6''-malonyl)glucoside-7-O-glucoside	Flavonols	26031.92
FBB73	Isovitexin-7-O-glucoside(Saponarin)	Flavones	3340
FBB74	Isovitexin-7-O-glucoside-2''-O-rhamnoside	Flavones	2775.81
FBB75	Kaempferol (3,5,7,4'-Tetrahydroxyflavone)	Flavonols	16843.49
FBB76	Kaempferol-3,7-O-diglucoside	Flavonols	69081.95
FBB77	Kaempferol-3-O-(2''-p-Coumaroyl)glucoside	Flavonols	16328.1
FBB78	Kaempferol-3-O-(6''-O-acetyl)glucoside	Flavonols	12065.36
FBB79	Kaempferol-3-O-(6''-p-coumaroyl)glucosyl-(1→2)-rhamnoside	Flavonols	12389.38
FBB80	Camelliaside A	Flavonols	8574.325
FBB81	Kaempferol-3-O-galactoside (Trifolin)*	Flavonols	1556796
FBB82	Kaempferol-3-O-galactoside-4'-O-glucoside	Flavonols	344990.7
FBB83	Kaempferol-3-O-glucoside (Astragalin)*	Flavonols	1217509
FBB84	Kaempferol-3-O-glucoside-7-O-rhamnoside*	Flavonols	6822474
FBB85	Kaempferol-3-O-glucuronide*	Flavonols	132705
FBB86	Kaempferol-3-O-neohesperidoside*	Flavonols	5977739
FBB87	Kaempferol-3-O-robinobioside(Biorobin)	Flavonols	1814777
FBB88	Kaempferol-3-O-rutinoside(Nicotiflorin)*	Flavonols	7250438
FBB89	Kaempferol-3-O-rutinoside-7-O-glucoside	Flavonols	329385.1
FBB90	Kaempferol-3-O-rutinoside-7-O-rhamnoside*	Flavonols	1147559
FBB91	Kaempferol-4'-O-glucoside*	Flavonols	1107830
FBB92	Kaempferol-7-O-glucoside*	Flavonols	1064745
FBB93	Luteolin-3'-O-glucoside	Flavones	6858552
FBB94	Luteolin-4'-O-glucoside	Flavones	6122888
FBB95	Luteolin-6-C-glucoside (Isoorientin)*	Flavones	5674485

ID	Compounds	Class II	Relative content （Area）
FBB96	Luteolin-7,3'-di-O-glucoside	Flavones	1823697
FBB97	Luteolin-7-O-gentiobioside	Flavones	531978.5
FBB98	Luteolin-7-O-glucoside (Cynaroside)*	Flavones	5117.224
FBB99	Luteolin-7-O-glucuronide*	Flavones	132705
FBB100	Luteolin-7-O-neohesperidoside (Lonicerin)*	Flavones	5741592
FBB101	Luteolin-7-O-rutinoside*	Flavones	975806
FBB102	Meratin*	Flavones	454108
FBB103	Monohydroxy-trimethoxyflavone-O-(6''-malonyl)glucoside	Flavones	10005
FBB104	Morin*	Flavonols	1349372
FBB105	Morin-3-O-xyloside*	Flavonols	49438.15
FBB106	Naringenin-7-O-Rutinoside-5-O-glucoside	Flavanones	78509.07
FBB107	Neosakuranin	Flavanones	992954.2
FBB108	Nigrolineaxanthone K	Other Flavonoids	108284.1
FBB109	Norartocarpetin	Flavones	737119.5
FBB110	Okanin-3',4'-di-O-glucoside	Chalcones	15723.23
FBB111	Okanin-3'-O-β-D-glucoside*	Chalcones	212748
FBB112	Okanin-4'-O-gentiobioside	Chalcones	13623.48
FBB113	Okanin-4'-O-glucoside(Marein)*	Chalcones	212748
FBB114	O-MethylNaringenin-8-C-arabinoside	Flavanones	111005.4
FBB115	Orientin-7-O-arabinoside	Flavones	12883.21
FBB116	Orientin-7-O-glucoside*	Flavones	369307.3
FBB117	Oroxin A	Flavones	124523.7
FBB118	Phellodendroside	Flavanonols	18582.09
FBB119	Phloretin	Chalcones	55683.46
FBB120	Prunetin-4'-O-glucoside(Prunitrin)	Isoflavones	92054.54
FBB121	Quercetin	Flavonols	102913.3
FBB122	Quercetin-3,3'-dimethyl ether	Flavonols	113624.1
FBB123	Quercetin-3,7-Di-O-glucoside*	Flavonols	420874.2
FBB124	Quercetin-3-O-(2''-O-galactosyl)glucoside	Flavonols	408554.5
FBB125	Quercetin-3-O-(2''-O-glucosyl)glucuronide	Flavonols	27211.13
FBB126	Quercetin-3-O-(2''-O-Rhamnosyl)rutinoside	Flavonols	1210503
FBB127	Quercetin-3-O-(2''-O-Xylosyl)rutinoside	Flavonols	78509.07
FBB128	Quercetin-3-O-[2''-O-(6'''-p-coumaroyl)glucosy1]rhamnoside	Flavonols	524499.1
FBB129	Quercetin3-O-galactoside*	Flavonols	6661558
FBB130	Quercetin-3-O-glucoside (Isoquercitrin)*	Flavonols	7275207
FBB131	Quercetin-3-O-glucoside-7-O-rhamnoside*	Flavonols	12037842
FBB132	Quercetin-3-O-glucuronide	Flavonols	1261713

ID	Compounds	Class II	Relative content （Area）
FBB133	Quercetin-3-O-neohesperidoside*	Flavonols	9711869
FBB134	Quercetin-3-O-robinobioside	Flavonols	1860153
FBB135	Quercetin-3-O-rutinoside (Rutin)*	Flavonols	10554023
FBB136	Quercetin-3-O-rutinoside-7-O-glucoside*	Flavonols	807238.1
FBB137	Quercetin-3-O-rutinoside-7-O-rhamnoside	Flavonols	445585
FBB138	Quercetin-3-O-Sambubioside-5-O-Glucoside	Flavonols	28441.2
FBB139	Quercetin-3-O-sophoroside (Baimaside)	Flavonols	40306.2
FBB140	Quercetin-3-O-xyloside (Reynoutrin)*	Flavonols	65537.83
FBB141	Quercetin-3-O-α-rhamnosyl (1→2)-[α-rhamnosyl (1→6)]-β-glucoside	Flavonols	1252029
FBB142	Quercetin-4'-O-glucoside (Spiraeoside)*	Flavonols	2432499
FBB143	Quercetin-4'-O-glucuronide*	Flavonols	7998574
FBB144	Quercetin-5-O-glucuronide*	Flavonols	7750188
FBB145	Quercetin-5-O-β-D-glucoside*	Flavonols	5537863
FBB146	Quercetin-7-O-glucoside	Flavonols	2186546
FBB147	Quercetin-7-O-rutinoside*	Flavonols	8420061
FBB148	Sachaloside IV*	Flavones	807238.1
FBB149	Sexangularetin	Flavonols	22654.08
FBB150	Sterubin 5-O-Glucoside	Flavanones	8066496
FBB151	Swertisin	Flavones	29793.43
FBB152	Syringetin-3-O-rutinoside-7-O-glucoside	Flavonols	53571.91
FBB153	Taxifolin(Dihydroquercetin)	Flavanonols	237209.5
FBB154	Thymonin (5,6,4'-Trihydroxy-7,8,3'-trimethoxyflavone)	Flavones	3498.327
FBB155	Tricetin 3'-glucuronide*	Flavones	9257413
FBB156	Tricin-4'-O-(guaiacylglycerol)ether*	Flavones	9942.394
FBB157	Tricin-4'-O-eudesmic acid	Flavones	17764.7
FBB158	Tricin-5-O-arabinoside	Flavones	9056.65
FBB159	Tricin-5-O-guaiacylglycerol*	Flavones	7749.539
FBB160	Tricin-7-O-guaiacylglycerol*	Flavones	9993.282
FBB161	Tricin-7-O-saccharic acid	Flavones	753964.9
FBB162	Vaccarin	Flavones	53571.91
FBB163	Vanillyl-O-Vitexin	Flavones	70056.25

Table S2

P value for compound-target interaction analysis

ID	Compounds	gene	Probability
FBB13	Chrysin	*ABCB1*	1
FBB13	Chrysin	*CA12*	1
FBB13	Chrysin	*CA4*	1
FBB13	Chrysin	*CDK5*	1
FBB13	Chrysin	*CDK6*	1
FBB13	Chrysin	*XDH*	1
FBB12	Kaempferol	*ABCB1*	1
FBB12	Kaempferol	*ABCC1*	1
FBB12	Kaempferol	*ALOX5*	1
FBB12	Kaempferol	*CA12*	1
FBB12	Kaempferol	*FLT3*	1
FBB12	Kaempferol	*XDH*	1
FBB15	Quercetin	*ABCB1*	1
FBB15	Quercetin	*ABCC1*	1
FBB15	Quercetin	*ADORA1*	1
FBB15	Quercetin	*ADORA2A*	1
FBB15	Quercetin	*AKR1A1*	1
FBB15	Quercetin	*AKR1C2*	1
FBB15	Quercetin	*AKT1*	1
FBB15	Quercetin	*ALK*	1
FBB15	Quercetin	*ALOX5*	1
FBB15	Quercetin	*AXL*	1
FBB15	Quercetin	*CA12*	1
FBB15	Quercetin	*CA4*	1
FBB15	Quercetin	*CA5A*	1
FBB15	Quercetin	*CXCR1*	1
FBB15	Quercetin	*DAPK1*	1
FBB15	Quercetin	*EGFR*	1
FBB15	Quercetin	*F2*	1
FBB15	Quercetin	*FLT3*	1
FBB15	Quercetin	*GPR35*	1
FBB15	Quercetin	*GSK3B*	1
FBB15	Quercetin	*IGF1R*	1
FBB15	Quercetin	*KDR*	1
FBB15	Quercetin	*MET*	1
FBB15	Quercetin	*MMP2*	1
FBB15	Quercetin	*MMP3*	1
FBB15	Quercetin	*MMP9*	1
FBB15	Quercetin	*MPO*	1
FBB15	Quercetin	*PIK3R1*	1
FBB15	Quercetin	*PIM1*	1
FBB15	Quercetin	*PLA2G1B*	1
FBB15	Quercetin	*PTK2*	1
FBB15	Quercetin	*SRC*	1
FBB15	Quercetin	*XDH*	1

ID	Compounds	gene	Probability
FBB38	7-Hydroxyflavone	*ADORA1*	0.982961
FBB38	7-Hydroxyflavone	*ADORA2A*	0.982961
FBB38	7-Hydroxyflavone	*CA12*	0.982961
FBB157	Gossypetin	*PIM1*	0.959682
FBB12	Kaempferol	*ADORA1*	0.795047
FBB12	Kaempferol	*CA4*	0.795047
FBB12	Kaempferol	*ACHE*	0.768469
FBB15	Quercetin	*ACHE*	0.680284
FBB15	Quercetin	*INSR*	0.680284
FBB15	Quercetin	*MAPT*	0.680284
FBB15	Quercetin	*MYLK*	0.680284
FBB15	Quercetin	*TOP2A*	0.680284
FBB21	2',3',4',5,7-Pentahydroxyflavone*	*ADORA1*	0.672321
FBB21	2',3',4',5,7-Pentahydroxyflavone*	*ALOX5*	0.672321
FBB21	2',3',4',5,7-Pentahydroxyflavone*	*CA12*	0.672321
FBB21	2',3',4',5,7-Pentahydroxyflavone*	*CA4*	0.672321
FBB21	2',3',4',5,7-Pentahydroxyflavone*	*FLT3*	0.672321
FBB21	2',3',4',5,7-Pentahydroxyflavone*	*GSK3B*	0.672321
FBB21	2',3',4',5,7-Pentahydroxyflavone*	*MMP2*	0.672321
FBB21	2',3',4',5,7-Pentahydroxyflavone*	*MMP9*	0.672321
FBB21	2',3',4',5,7-Pentahydroxyflavone*	*XDH*	0.672321
FBB12	Kaempferol	*GSK3B*	0.65802
FBB12	Kaempferol	*MMP2*	0.65802
FBB12	Kaempferol	*MMP9*	0.65802
FBB122	Norartocarpetin	*ABCC1*	0.616847
FBB122	Norartocarpetin	*ADORA1*	0.616847
FBB122	Norartocarpetin	*CDK5*	0.616847
FBB122	Norartocarpetin	*FLT3*	0.616847
FBB122	Norartocarpetin	*GSK3B*	0.616847
FBB122	Norartocarpetin	*TTR*	0.616847
FBB122	Norartocarpetin	*XDH*	0.616847
FBB12	Kaempferol	*PTPRS*	0.608446
FBB21	2',3',4',5,7-Pentahydroxyflavone*	*PTPRS*	0.577582
FBB12	Kaempferol	*ADORA2A*	0.567302
FBB151	Isobavachin	*ESR1*	0.556861
FBB151	Isobavachin	*ESR2*	0.556861
FBB21	2',3',4',5,7-Pentahydroxyflavone*	*ABCB1*	0.553794
FBB21	2',3',4',5,7-Pentahydroxyflavone*	*ABCC1*	0.553794
FBB21	2',3',4',5,7-Pentahydroxyflavone*	*ADORA2A*	0.553794
FBB21	2',3',4',5,7-Pentahydroxyflavone*	*AKR1A1*	0.553794
FBB21	2',3',4',5,7-Pentahydroxyflavone*	*AKR1C2*	0.553794
FBB21	2',3',4',5,7-Pentahydroxyflavone*	*AKT1*	0.553794
FBB21	2',3',4',5,7-Pentahydroxyflavone*	*ALK*	0.553794
FBB21	2',3',4',5,7-Pentahydroxyflavone*	*AXL*	0.553794
FBB21	2',3',4',5,7-Pentahydroxyflavone*	*CA5A*	0.553794
FBB21	2',3',4',5,7-Pentahydroxyflavone*	*CXCR1*	0.553794
FBB21	2',3',4',5,7-Pentahydroxyflavone*	*DAPK1*	0.553794
FBB21	2',3',4',5,7-Pentahydroxyflavone*	*EGFR*	0.553794
FBB21	2',3',4',5,7-Pentahydroxyflavone*	*F2*	0.553794

ID	Compounds	gene	Probability
FBB21	2',3',4',5,7-Pentahydroxyflavone*	*IGF1R*	0.553794
FBB21	2',3',4',5,7-Pentahydroxyflavone*	*KDR*	0.553794
FBB21	2',3',4',5,7-Pentahydroxyflavone*	*MET*	0.553794
FBB21	2',3',4',5,7-Pentahydroxyflavone*	*MMP3*	0.553794
FBB21	2',3',4',5,7-Pentahydroxyflavone*	*MPO*	0.553794
FBB21	2',3',4',5,7-Pentahydroxyflavone*	*PIK3R1*	0.553794
FBB21	2',3',4',5,7-Pentahydroxyflavone*	*PIM1*	0.553794
FBB21	2',3',4',5,7-Pentahydroxyflavone*	*PLA2G1B*	0.553794
FBB21	2',3',4',5,7-Pentahydroxyflavone*	*PTK2*	0.553794
FBB21	2',3',4',5,7-Pentahydroxyflavone*	*SRC*	0.553794
FBB157	Gossypetin	*ABCC1*	0.551553
FBB157	Gossypetin	*ACHE*	0.551553
FBB157	Gossypetin	*FLT3*	0.551553
FBB157	Gossypetin	*GPR35*	0.551553
FBB157	Gossypetin	*INSR*	0.551553
FBB157	Gossypetin	*MAPT*	0.551553
FBB157	Gossypetin	*MYLK*	0.551553
FBB157	Gossypetin	*TOP2A*	0.551553
FBB157	Gossypetin	*XDH*	0.551553
FBB15	Quercetin	*ESR2*	0.545872
FBB15	Quercetin	*PTPRS*	0.545872
FBB15	Quercetin	*SLC22A12*	0.545872
FBB15	Quercetin	*CDK5*	0.538062
FBB65	3-O-Methylquercetin	*APP*	0.526533
FBB122	Norartocarpetin	*ABCB1*	0.526222
FBB122	Norartocarpetin	*ALOX5*	0.526222
FBB122	Norartocarpetin	*CA12*	0.526222
FBB12	Kaempferol	*CDK5*	0.517921
FBB65	3-O-Methylquercetin	*XDH*	0.501556
FBB12	Kaempferol	*DAPK1*	0.50153
FBB12	Kaempferol	*ESR2*	0.50153
FBB12	Kaempferol	*GPR35*	0.50153
FBB12	Kaempferol	*SLC22A12*	0.50153
FBB15	Quercetin	*CDK6*	0.498513
FBB21	2',3',4',5,7-Pentahydroxyflavone*	*GPR35*	0.490609
FBB17	7,4'-Dihydroxydihydroflavonoids	*ESR1*	0.489174
FBB17	7,4'-Dihydroxydihydroflavonoids	*ESR2*	0.489174
FBB12	Kaempferol	*TTR*	0.484896
FBB122	Norartocarpetin	*ACHE*	0.484896
FBB122	Norartocarpetin	*CA4*	0.484896
FBB122	Norartocarpetin	*CDK6*	0.484896
FBB12	Kaempferol	*CDK6*	0.476836
FBB122	Norartocarpetin	*PTPRS*	0.468553
FBB65	3-O-Methylquercetin	*CA12*	0.468148
FBB65	3-O-Methylquercetin	*CA4*	0.468148
FBB17	7,4'-Dihydroxydihydroflavonoids	*ABCC1*	0.457782
FBB17	7,4'-Dihydroxydihydroflavonoids	*CA12*	0.457782
FBB17	7,4'-Dihydroxydihydroflavonoids	*CA4*	0.457782
FBB18	4'-Hydroxy-5,7-dimethoxyflavanone	*ADORA1*	0.443235

ID	Compounds	gene	Probability
FBB29	Mimulone	*ACHE*	0.431039
FBB157	Gossypetin	*DAPK1*	0.426503
FBB102	3-Methylkaempferol	*ABCB1*	0.419571
FBB21	2',3',4',5,7-Pentahydroxyflavone*	*ESR2*	0.419571
FBB21	2',3',4',5,7-Pentahydroxyflavone*	*SLC22A12*	0.419571
FBB13	Chrysin	*ESR1*	0.418443
FBB13	Chrysin	*ESR2*	0.418443
FBB12	Kaempferol	*AKR1A1*	0.402643
FBB12	Kaempferol	*AKR1C2*	0.402643
FBB12	Kaempferol	*AKT1*	0.402643
FBB12	Kaempferol	*ALK*	0.402643
FBB12	Kaempferol	*AXL*	0.402643
FBB12	Kaempferol	*CA5A*	0.402643
FBB12	Kaempferol	*CXCR1*	0.402643
FBB12	Kaempferol	*EGFR*	0.402643
FBB12	Kaempferol	*F2*	0.402643
FBB12	Kaempferol	*IGF1R*	0.402643
FBB12	Kaempferol	*KDR*	0.402643
FBB12	Kaempferol	*MET*	0.402643
FBB12	Kaempferol	*MMP3*	0.402643
FBB12	Kaempferol	*MPO*	0.402643
FBB12	Kaempferol	*PIK3R1*	0.402643
FBB12	Kaempferol	*PIM1*	0.402643
FBB12	Kaempferol	*PLA2G1B*	0.402643
FBB12	Kaempferol	*PTK2*	0.402643
FBB12	Kaempferol	*SRC*	0.402643
FBB122	Norartocarpetin	*APP*	0.402643
FBB122	Norartocarpetin	*CD38*	0.402643
FBB122	Norartocarpetin	*DAPK1*	0.402643
FBB122	Norartocarpetin	*ESR2*	0.402643
FBB122	Norartocarpetin	*GPR35*	0.402643
FBB122	Norartocarpetin	*MMP12*	0.402643
FBB122	Norartocarpetin	*MMP2*	0.402643
FBB122	Norartocarpetin	*MMP9*	0.402643
FBB122	Norartocarpetin	*SLC22A12*	0.402643
FBB122	Norartocarpetin	*TOP1*	0.402643
FBB157	Gossypetin	*ABCB1*	0.376566
FBB157	Gossypetin	*ADORA1*	0.376566
FBB157	Gossypetin	*ADORA2A*	0.376566
FBB157	Gossypetin	*AKR1A1*	0.376566
FBB157	Gossypetin	*AKR1C2*	0.376566
FBB157	Gossypetin	*AKT1*	0.376566
FBB157	Gossypetin	*ALK*	0.376566
FBB157	Gossypetin	*ALOX5*	0.376566
FBB157	Gossypetin	*AXL*	0.376566
FBB157	Gossypetin	*CA12*	0.376566
FBB157	Gossypetin	*CA4*	0.376566
FBB157	Gossypetin	*CA5A*	0.376566
FBB157	Gossypetin	*CXCR1*	0.376566

ID	Compounds	gene	Probability
FBB157	Gossypetin	*EGFR*	0.376566
FBB157	Gossypetin	*F2*	0.376566
FBB157	Gossypetin	*GSK3B*	0.376566
FBB157	Gossypetin	*IGF1R*	0.376566
FBB157	Gossypetin	*KDR*	0.376566
FBB157	Gossypetin	*MET*	0.376566
FBB157	Gossypetin	*MMP2*	0.376566
FBB157	Gossypetin	*MMP3*	0.376566
FBB157	Gossypetin	*MMP9*	0.376566
FBB157	Gossypetin	*MPO*	0.376566
FBB157	Gossypetin	*PIK3R1*	0.376566
FBB157	Gossypetin	*PLA2G1B*	0.376566
FBB157	Gossypetin	*PTK2*	0.376566
FBB157	Gossypetin	*SRC*	0.376566
FBB13	Chrysin	*ACHE*	0.371345
FBB13	Chrysin	*ADORA1*	0.371345
FBB13	Chrysin	*ADORA2A*	0.371345
FBB21	2',3',4',5,7-Pentahydroxyflavone*	*APP*	0.364128
FBB21	2',3',4',5,7-Pentahydroxyflavone*	*CD38*	0.364128
FBB21	2',3',4',5,7-Pentahydroxyflavone*	*CDK5*	0.364128
FBB21	2',3',4',5,7-Pentahydroxyflavone*	*MMP12*	0.364128
FBB21	2',3',4',5,7-Pentahydroxyflavone*	*TOP1*	0.364128
FBB21	2',3',4',5,7-Pentahydroxyflavone*	*TTR*	0.364128
FBB102	3-Methylkaempferol	*ADORA1*	0.356368
FBB13	Chrysin	*GRK6*	0.347652
FBB11	Quercetin-3,3'-dimethyl ether	*CA12*	0.346958
FBB11	Quercetin-3,3'-dimethyl ether	*CA4*	0.346958
FBB11	Quercetin-3,3'-dimethyl ether	*XDH*	0.346958
FBB46	Eupatorin-5-methylether	*ALOX5*	0.34463
FBB123	Morin	*DAPK1*	0.340526
FBB123	Morin	*ESR2*	0.340526
FBB123	Morin	*PTPRS*	0.340526
FBB123	Morin	*SLC22A12*	0.340526
FBB122	Norartocarpetin	*ADORA2A*	0.33674
FBB122	Norartocarpetin	*CFTR*	0.328396
FBB122	Norartocarpetin	*ESR1*	0.328396
FBB122	Norartocarpetin	*PTGS2*	0.328396
FBB18	4'-Hydroxy-5,7-dimethoxyflavanone	*PTGS1*	0.316876
FBB21	2',3',4',5,7-Pentahydroxyflavone*	*ACHE*	0.316876
FBB21	2',3',4',5,7-Pentahydroxyflavone*	*INSR*	0.316876
FBB21	2',3',4',5,7-Pentahydroxyflavone*	*MAPT*	0.316876
FBB21	2',3',4',5,7-Pentahydroxyflavone*	*MYLK*	0.316876
FBB21	2',3',4',5,7-Pentahydroxyflavone*	*TOP2A*	0.316876
FBB18	4'-Hydroxy-5,7-dimethoxyflavanone	*ESR1*	0.308961
FBB18	4'-Hydroxy-5,7-dimethoxyflavanone	*ADORA3*	0.30103
FBB12	Kaempferol	*APP*	0.295453
FBB12	Kaempferol	*CD38*	0.295453
FBB12	Kaempferol	*MMP12*	0.295453

ID	Compounds	gene	Probability
FBB12	Kaempferol	*TOP1*	0.295453
FBB102	3-Methylkaempferol	*ABCC1*	0.293176
FBB38	7-Hydroxyflavone	*ESR2*	0.289501
FBB65	3-O-Methylquercetin	*ABCC1*	0.284878
FBB102	3-Methylkaempferol	*ADORA2A*	0.277371
FBB13	Chrysin	*ABCC1*	0.276999
FBB13	Chrysin	*CFTR*	0.276999
FBB13	Chrysin	*FLT3*	0.276999
FBB13	Chrysin	*GSK3B*	0.276999
FBB13	Chrysin	*PTGS2*	0.276999
FBB13	Chrysin	*TTR*	0.276999
FBB65	3-O-Methylquercetin	*PLG*	0.276589
FBB12	Kaempferol	*CFTR*	0.270837
FBB12	Kaempferol	*ESR1*	0.270837
FBB12	Kaempferol	*PTGS2*	0.270837
FBB12	Kaempferol	*PFKFB3*	0.262582
FBB15	Quercetin	*APP*	0.26156
FBB15	Quercetin	*CD38*	0.26156
FBB15	Quercetin	*MMP12*	0.26156
FBB15	Quercetin	*TOP1*	0.26156
FBB15	Quercetin	*TTR*	0.26156
FBB65	3-O-Methylquercetin	*ABCB1*	0.25991
FBB29	Mimulone	*PPARG*	0.256112
FBB18	4'-Hydroxy-5,7-dimethoxyflavanone	*CA12*	0.253687
FBB17	7,4'-Dihydroxydihydroflavonoids	*PTGS1*	0.253454
FBB157	Gossypetin	*PTPRS*	0.251529
FBB11	Quercetin-3,3'-dimethyl ether	*APP*	0.250033
FBB122	Norartocarpetin	*GRK6*	0.246151
FBB13	Chrysin	*LCK*	0.245481
FBB38	7-Hydroxyflavone	*TTR*	0.244186
FBB12	Kaempferol	*GRK6*	0.237885
FBB65	3-O-Methylquercetin	*ADORA1*	0.234886
FBB65	3-O-Methylquercetin	*EGFR*	0.234886
FBB65	3-O-Methylquercetin	*IGF1R*	0.234886
FBB2	Nigrolineaxanthone K	*RARA*	0.23112
FBB12	Kaempferol	*TERT*	0.229686
FBB11	Quercetin-3,3'-dimethyl ether	*PLG*	0.225803
FBB29	Mimulone	*ESR1*	0.222798
FBB29	Mimulone	*ESR2*	0.222798
FBB18	4'-Hydroxy-5,7-dimethoxyflavanone	*ABCC1*	0.214179
FBB21	2',3',4',5,7-Pentahydroxyflavone*	*CDK6*	0.214179
FBB123	Morin	*ABCB1*	0.214179
FBB123	Morin	*ABCC1*	0.214179
FBB123	Morin	*ALOX5*	0.214179
FBB123	Morin	*FLT3*	0.214179
FBB123	Morin	*XDH*	0.214179
FBB17	7,4'-Dihydroxydihydroflavonoids	*ADORA1*	0.21416
FBB17	7,4'-Dihydroxydihydroflavonoids	*ADORA3*	0.21416

ID	Compounds	gene	Probability
FBB161	Artemetin	*XDH*	0.21412
FBB38	7-Hydroxyflavone	*ABCB1*	0.207866
FBB38	7-Hydroxyflavone	*CA4*	0.207866
FBB38	7-Hydroxyflavone	*CDK5*	0.207866
FBB38	7-Hydroxyflavone	*CDK6*	0.207866
FBB38	7-Hydroxyflavone	*XDH*	0.207866
FBB157	Gossypetin	*PFKFB3*	0.201602
FBB65	3-O-Methylquercetin	*ACHE*	0.201602
FBB16	Ranupetin	*XDH*	0.201585
FBB46	Eupatorin-5-methylether	*KIT*	0.19862
FBB13	Chrysin	*IKBKB*	0.198444
FBB18	4'-Hydroxy-5,7-dimethoxyflavanone	*ESR2*	0.198389
FBB102	3-Methylkaempferol	*APP*	0.198389
FBB102	3-Methylkaempferol	*PLG*	0.198389
FBB15	Quercetin	*TERT*	0.198389
FBB66	Sexangularetin	*ADORA1*	0.19327
FBB66	Sexangularetin	*APP*	0.19327
FBB102	3-Methylkaempferol	*CA12*	0.190502
FBB102	3-Methylkaempferol	*XDH*	0.190502
FBB66	Sexangularetin	*NOS2*	0.18493
FBB65	3-O-Methylquercetin	*ADORA2A*	0.18493
FBB65	3-O-Methylquercetin	*AKR1A1*	0.18493
FBB65	3-O-Methylquercetin	*AKR1C2*	0.18493
FBB65	3-O-Methylquercetin	*AKT1*	0.18493
FBB65	3-O-Methylquercetin	*ALK*	0.18493
FBB65	3-O-Methylquercetin	*ALOX5*	0.18493
FBB65	3-O-Methylquercetin	*AXL*	0.18493
FBB65	3-O-Methylquercetin	*CA5A*	0.18493
FBB65	3-O-Methylquercetin	*CXCR1*	0.18493
FBB65	3-O-Methylquercetin	*DAPK1*	0.18493
FBB65	3-O-Methylquercetin	*F2*	0.18493
FBB65	3-O-Methylquercetin	*FLT3*	0.18493
FBB65	3-O-Methylquercetin	*GSK3B*	0.18493
FBB65	3-O-Methylquercetin	*KDR*	0.18493
FBB65	3-O-Methylquercetin	*MET*	0.18493
FBB65	3-O-Methylquercetin	*MMP2*	0.18493
FBB65	3-O-Methylquercetin	*MMP3*	0.18493
FBB65	3-O-Methylquercetin	*MMP9*	0.18493
FBB65	3-O-Methylquercetin	*MPO*	0.18493
FBB65	3-O-Methylquercetin	*PIK3R1*	0.18493
FBB65	3-O-Methylquercetin	*PIM1*	0.18493
FBB65	3-O-Methylquercetin	*PLA2G1B*	0.18493
FBB65	3-O-Methylquercetin	*PTK2*	0.18493
FBB65	3-O-Methylquercetin	*SRC*	0.18493
FBB13	Chrysin	*ADORA3*	0.182723
FBB46	Eupatorin-5-methylether	*ADORA2A*	0.182369
FBB16	Ranupetin	*PIM1*	0.177363
FBB151	Isobavachin	*ACHE*	0.177363
FBB66	Sexangularetin	*EGFR*	0.176594

ID	Compounds	gene	Probability
FBB157	Gossypetin	*TERT*	0.176594
FBB65	3-O-Methylquercetin	*GPR35*	0.176594
FBB18	4'-Hydroxy-5,7-dimethoxyflavanone	*CA4*	0.174646
FBB102	3-Methylkaempferol	*ALOX5*	0.174646
FBB102	3-Methylkaempferol	*FLT3*	0.174646
FBB21	2',3',4',5,7-Pentahydroxyflavone*	*ESR1*	0.174646
FBB12	Kaempferol	*MAPT*	0.171979
FBB66	Sexangularetin	*ADORA2A*	0.168265
FBB66	Sexangularetin	*PIM1*	0.168265
FBB13	Chrysin	*ALOX5*	0.167042
FBB13	Chrysin	*PIM1*	0.167042
FBB102	3-Methylkaempferol	*ADORA3*	0.166799
FBB123	Morin	*ACHE*	0.166799
FBB123	Morin	*ADORA1*	0.166799
FBB123	Morin	*ADORA2A*	0.166799
FBB123	Morin	*ADORA3*	0.166799
FBB123	Morin	*GPR35*	0.166799
FBB161	Artemetin	*CA12*	0.164912
FBB11	Quercetin-3,3'-dimethyl ether	*ABCC1*	0.161187
FBB16	Ranupetin	*ADORA3*	0.161187
FBB13	Chrysin	*EGFR*	0.159189
FBB13	Chrysin	*PTPRS*	0.159189
FBB102	3-Methylkaempferol	*ACHE*	0.158886
FBB102	3-Methylkaempferol	*CA4*	0.158886
FBB19	Thymonin	*ALOX5*	0.158035
FBB46	Eupatorin-5-methylether	*ABCC1*	0.158035
FBB46	Eupatorin-5-methylether	*ADORA1*	0.158035
FBB46	Eupatorin-5-methylether	*ADORA3*	0.158035
FBB122	Norartocarpetin	*AKR1A1*	0.155528
FBB122	Norartocarpetin	*AKR1C2*	0.155528
FBB122	Norartocarpetin	*AKT1*	0.155528
FBB122	Norartocarpetin	*ALK*	0.155528
FBB122	Norartocarpetin	*AXL*	0.155528
FBB122	Norartocarpetin	*CA5A*	0.155528
FBB122	Norartocarpetin	*CXCR1*	0.155528
FBB122	Norartocarpetin	*EGFR*	0.155528
FBB122	Norartocarpetin	*F2*	0.155528
FBB122	Norartocarpetin	*IGF1R*	0.155528
FBB122	Norartocarpetin	*KDR*	0.155528
FBB122	Norartocarpetin	*MET*	0.155528
FBB122	Norartocarpetin	*MMP3*	0.155528
FBB122	Norartocarpetin	*MPO*	0.155528
FBB122	Norartocarpetin	*PIK3R1*	0.155528
FBB122	Norartocarpetin	*PIM1*	0.155528
FBB122	Norartocarpetin	*PLA2G1B*	0.155528
FBB122	Norartocarpetin	*PTK2*	0.155528
FBB122	Norartocarpetin	*SRC*	0.155528
FBB16	Ranupetin	*EGFR*	0.153093

ID	Compounds	gene	Probability
FBB43	(3S)-5,7-dihydroxy-3-[(4-methoxyphenyl)methyl]-6-methyl-2,3-dihydrochromen-4-one*	*ESR1*	0.151565
FBB157	Gossypetin	*CDK5*	0.151565
FBB157	Gossypetin	*ESR2*	0.151565
FBB157	Gossypetin	*SLC22A12*	0.151565
FBB65	3-O-Methylquercetin	*CDK5*	0.151565
FBB65	3-O-Methylquercetin	*CDK6*	0.151565
FBB13	Chrysin	*AR*	0.151315
FBB102	3-Methylkaempferol	*EGFR*	0.150982
FBB102	3-Methylkaempferol	*IGF1R*	0.150982
FBB16	Ranupetin	*ADORA1*	0.145047
FBB16	Ranupetin	*ADORA2A*	0.145047
FBB16	Ranupetin	*CA12*	0.145047
FBB16	Ranupetin	*CA4*	0.145047
FBB38	7-Hydroxyflavone	*ACHE*	0.144331
FBB38	7-Hydroxyflavone	*ESR1*	0.144331
FBB38	7-Hydroxyflavone	*FLT3*	0.144331
FBB38	7-Hydroxyflavone	*GRK6*	0.144331
FBB125	3-O-Acetylpinobanksin	*MET*	0.143269
FBB65	3-O-Methylquercetin	*INSR*	0.143269
FBB65	3-O-Methylquercetin	*MAPT*	0.143269
FBB65	3-O-Methylquercetin	*MYLK*	0.143269
FBB65	3-O-Methylquercetin	*TOP2A*	0.143269
FBB123	Morin	*AKR1A1*	0.143102
FBB123	Morin	*AKR1C2*	0.143102
FBB123	Morin	*ALK*	0.143102
FBB123	Morin	*AXL*	0.143102
FBB123	Morin	*CXCR1*	0.143102
FBB123	Morin	*MAPT*	0.143102
FBB123	Morin	*MMP2*	0.143102
FBB123	Morin	*MMP3*	0.143102
FBB123	Morin	*MMP9*	0.143102
FBB123	Morin	*MPO*	0.143102
FBB123	Morin	*MYLK*	0.143102
FBB123	Morin	*PIK3R1*	0.143102
FBB123	Morin	*PIM1*	0.143102
FBB123	Morin	*PLA2G1B*	0.143102
FBB123	Morin	*TOP2A*	0.143102
FBB52	3,5-Diacetyltambulin	*ADORA1*	0.143015
FBB46	Eupatorin-5-methylether	*PIM1*	0.141778
FBB161	Artemetin	*ADORA3*	0.140333
FBB161	Artemetin	*APP*	0.140333
FBB122	Norartocarpetin	*TERT*	0.139062
FBB11	Quercetin-3,3'-dimethyl ether	*ABCB1*	0.13697
FBB16	Ranupetin	*APP*	0.13697
FBB16	Ranupetin	*DAPK1*	0.13697
FBB16	Ranupetin	*GPR35*	0.13697
FBB16	Ranupetin	*TERT*	0.13697
FBB13	Chrysin	*APP*	0.135616

ID	Compounds	gene	Probability
FBB13	Chrysin	*PFKFB3*	0.135616
FBB38	7-Hydroxyflavone	*LCK*	0.135226
FBB9	2',7-Dihydroxy-3',4'-dimethoxyisoflavan	*ALOX15B*	0.135202
FBB102	3-Methylkaempferol	*ESR2*	0.135202
FBB102	3-Methylkaempferol	*PTPRS*	0.135202
FBB52	3,5-Diacetyltambulin	*ADORA3*	0.134972
FBB157	Gossypetin	*CDK6*	0.134939
FBB19	Thymonin	*TERT*	0.133684
FBB46	Eupatorin-5-methylether	*APP*	0.133684
FBB46	Eupatorin-5-methylether	*GSK3B*	0.133684
FBB161	Artemetin	*ADORA1*	0.132135
FBB161	Artemetin	*PLG*	0.132135
FBB29	Mimulone	*PLA2G1B*	0.131156
FBB16	Ranupetin	*ABCB1*	0.128899
FBB16	Ranupetin	*ABCC1*	0.128899
FBB16	Ranupetin	*ALOX5*	0.128899
FBB16	Ranupetin	*FLT3*	0.128899
FBB41	Eucalyptin	*ABCB1*	0.128899
FBB151	Isobavachin	*CA4*	0.128899
FBB13	Chrysin	*CD38*	0.12775
FBB13	Chrysin	*CYP1A1*	0.12775
FBB13	Chrysin	*MMP12*	0.12775
FBB13	Chrysin	*MMP2*	0.12775
FBB13	Chrysin	*MMP9*	0.12775
FBB13	Chrysin	*TERT*	0.12775
FBB13	Chrysin	*TOP1*	0.12775
FBB17	7,4'-Dihydroxydihydroflavonoids	*PLA2G1B*	0.12775
FBB17	7,4'-Dihydroxydihydroflavonoids	*PPARG*	0.12775
FBB18	4'-Hydroxy-5,7-dimethoxyflavanone	*PPARG*	0.127303
FBB102	3-Methylkaempferol	*CDK5*	0.127303
FBB102	3-Methylkaempferol	*CDK6*	0.127303
FBB102	3-Methylkaempferol	*DAPK1*	0.127303
FBB102	3-Methylkaempferol	*GPR35*	0.127303
FBB102	3-Methylkaempferol	*GSK3B*	0.127303
FBB102	3-Methylkaempferol	*MMP2*	0.127303
FBB102	3-Methylkaempferol	*MMP9*	0.127303
FBB102	3-Methylkaempferol	*PTGS2*	0.127303
FBB102	3-Methylkaempferol	*SLC22A12*	0.127303
FBB123	Morin	*CA12*	0.127303
FBB123	Morin	*CDK5*	0.127303
FBB123	Morin	*CDK6*	0.127303
FBB123	Morin	*PFKFB3*	0.127303
FBB66	Sexangularetin	*ESR2*	0.12661
FBB66	Sexangularetin	*PTGS2*	0.12661
FBB157	Gossypetin	*ADORA3*	0.12661
FBB157	Gossypetin	*ST6GAL1*	0.12661
FBB38	7-Hydroxyflavone	*IKBKB*	0.126154
FBB19	Thymonin	*OPRM1*	0.125572

ID	Compounds	gene	Probability
FBB19	Thymonin	*XDH*	0.125572
FBB46	Eupatorin-5-methylether	*MMP2*	0.125572
FBB46	Eupatorin-5-methylether	*MMP9*	0.125572
FBB161	Artemetin	*ADORA2A*	0.123937
FBB161	Artemetin	*ALOX5*	0.123937
FBB122	Norartocarpetin	*AR*	0.122582
FBB11	Quercetin-3,3'-dimethyl ether	*F2*	0.120824
FBB16	Ranupetin	*ACHE*	0.120824
FBB16	Ranupetin	*AKR1A1*	0.120824
FBB16	Ranupetin	*AKR1C2*	0.120824
FBB16	Ranupetin	*AKT1*	0.120824
FBB16	Ranupetin	*ALK*	0.120824
FBB16	Ranupetin	*AXL*	0.120824
FBB16	Ranupetin	*CA5A*	0.120824
FBB16	Ranupetin	*CXCR1*	0.120824
FBB16	Ranupetin	*F2*	0.120824
FBB16	Ranupetin	*GSK3B*	0.120824
FBB16	Ranupetin	*IGF1R*	0.120824
FBB16	Ranupetin	*INSR*	0.120824
FBB16	Ranupetin	*KDR*	0.120824
FBB16	Ranupetin	*MAPT*	0.120824
FBB16	Ranupetin	*MET*	0.120824
FBB16	Ranupetin	*MMP2*	0.120824
FBB16	Ranupetin	*MMP3*	0.120824
FBB16	Ranupetin	*MMP9*	0.120824
FBB16	Ranupetin	*MPO*	0.120824
FBB16	Ranupetin	*MYLK*	0.120824
FBB16	Ranupetin	*PIK3R1*	0.120824
FBB16	Ranupetin	*PLA2G1B*	0.120824
FBB16	Ranupetin	*PTK2*	0.120824
FBB16	Ranupetin	*SRC*	0.120824
FBB16	Ranupetin	*TOP2A*	0.120824
FBB151	Isobavachin	*PPARG*	0.120824
FBB13	Chrysin	*INSR*	0.119895
FBB13	Chrysin	*MAPT*	0.119895
FBB13	Chrysin	*MET*	0.119895
FBB13	Chrysin	*MYLK*	0.119895
FBB13	Chrysin	*SLC22A12*	0.119895
FBB13	Chrysin	*ST6GAL1*	0.119895
FBB13	Chrysin	*TOP2A*	0.119895
FBB17	7,4'-Dihydroxydihydroflavonoids	*MMP12*	0.119895
FBB18	4'-Hydroxy-5,7-dimethoxyflavanone	*MMP12*	0.119404
FBB18	4'-Hydroxy-5,7-dimethoxyflavanone	*PLA2G1B*	0.119404
FBB102	3-Methylkaempferol	*AKR1A1*	0.119404
FBB102	3-Methylkaempferol	*AKR1C2*	0.119404
FBB102	3-Methylkaempferol	*AKT1*	0.119404
FBB102	3-Methylkaempferol	*ALK*	0.119404
FBB102	3-Methylkaempferol	*AXL*	0.119404

ID	Compounds	gene	Probability
FBB102	3-Methylkaempferol	*CA5A*	0.119404
FBB102	3-Methylkaempferol	*CXCR1*	0.119404
FBB102	3-Methylkaempferol	*ESR1*	0.119404
FBB102	3-Methylkaempferol	*F2*	0.119404
FBB102	3-Methylkaempferol	*KDR*	0.119404
FBB102	3-Methylkaempferol	*MET*	0.119404
FBB102	3-Methylkaempferol	*MMP3*	0.119404
FBB102	3-Methylkaempferol	*MPO*	0.119404
FBB102	3-Methylkaempferol	*PFKFB3*	0.119404
FBB102	3-Methylkaempferol	*PIK3R1*	0.119404
FBB102	3-Methylkaempferol	*PIM1*	0.119404
FBB102	3-Methylkaempferol	*PLA2G1B*	0.119404
FBB102	3-Methylkaempferol	*PTK2*	0.119404
FBB102	3-Methylkaempferol	*SRC*	0.119404
FBB123	Morin	*CA4*	0.119404
FBB123	Morin	*CFTR*	0.119404
FBB123	Morin	*ESR1*	0.119404
FBB123	Morin	*F2*	0.119404
FBB123	Morin	*GRK6*	0.119404
FBB123	Morin	*PTGS2*	0.119404
FBB123	Morin	*TTR*	0.119404
FBB52	3,5-Diacetyltambulin	*ABCB1*	0.118883
FBB52	3,5-Diacetyltambulin	*ADORA2A*	0.118883
FBB52	3,5-Diacetyltambulin	*AGTR2*	0.118883
FBB52	3,5-Diacetyltambulin	*AKR1A1*	0.118883
FBB52	3,5-Diacetyltambulin	*AKR1C2*	0.118883
FBB52	3,5-Diacetyltambulin	*AKT1*	0.118883
FBB52	3,5-Diacetyltambulin	*ALK*	0.118883
FBB52	3,5-Diacetyltambulin	*ALOX5*	0.118883
FBB52	3,5-Diacetyltambulin	*APP*	0.118883
FBB52	3,5-Diacetyltambulin	*AXL*	0.118883
FBB52	3,5-Diacetyltambulin	*CA12*	0.118883
FBB52	3,5-Diacetyltambulin	*CCND1*	0.118883
FBB52	3,5-Diacetyltambulin	*CCNA1*	0.118883
FBB52	3,5-Diacetyltambulin	*CDK5*	0.118883
FBB52	3,5-Diacetyltambulin	*CRHR1*	0.118883
FBB52	3,5-Diacetyltambulin	*CSF1R*	0.118883
FBB52	3,5-Diacetyltambulin	*EGFR*	0.118883
FBB52	3,5-Diacetyltambulin	*F2*	0.118883
FBB52	3,5-Diacetyltambulin	*FLT3*	0.118883
FBB52	3,5-Diacetyltambulin	*FLT4*	0.118883
FBB52	3,5-Diacetyltambulin	*GSK3B*	0.118883
FBB52	3,5-Diacetyltambulin	*IGF1R*	0.118883
FBB52	3,5-Diacetyltambulin	*INSR*	0.118883
FBB52	3,5-Diacetyltambulin	*JAK2*	0.118883
FBB52	3,5-Diacetyltambulin	*KDR*	0.118883
FBB52	3,5-Diacetyltambulin	*KIT*	0.118883
FBB52	3,5-Diacetyltambulin	*MAPK8*	0.118883
FBB52	3,5-Diacetyltambulin	*MET*	0.118883

ID	Compounds	gene	Probability
FBB52	3,5-Diacetyltambulin	*MMP2*	0.118883
FBB52	3,5-Diacetyltambulin	*MMP9*	0.118883
FBB52	3,5-Diacetyltambulin	*MPO*	0.118883
FBB52	3,5-Diacetyltambulin	*NOS2*	0.118883
FBB52	3,5-Diacetyltambulin	*NR5A1*	0.118883
FBB52	3,5-Diacetyltambulin	*OPRM1*	0.118883
FBB52	3,5-Diacetyltambulin	*PDE10A*	0.118883
FBB52	3,5-Diacetyltambulin	*PDE4D*	0.118883
FBB52	3,5-Diacetyltambulin	*PDE5A*	0.118883
FBB52	3,5-Diacetyltambulin	*PDGFRB*	0.118883
FBB52	3,5-Diacetyltambulin	*PDGFRB*	0.118883
FBB52	3,5-Diacetyltambulin	*PIK3CA*	0.118883
FBB52	3,5-Diacetyltambulin	*PIK3R1*	0.118883
FBB52	3,5-Diacetyltambulin	*PLA2G1B*	0.118883
FBB52	3,5-Diacetyltambulin	*PLG*	0.118883
FBB52	3,5-Diacetyltambulin	*PTGS2*	0.118883
FBB52	3,5-Diacetyltambulin	*PTK2*	0.118883
FBB52	3,5-Diacetyltambulin	*PTK2B*	0.118883
FBB52	3,5-Diacetyltambulin	*ROCK1*	0.118883
FBB52	3,5-Diacetyltambulin	*ROS1*	0.118883
FBB52	3,5-Diacetyltambulin	*SRC*	0.118883
FBB52	3,5-Diacetyltambulin	*TACR1*	0.118883
FBB52	3,5-Diacetyltambulin	*TERT*	0.118883
FBB52	3,5-Diacetyltambulin	*TOP2A*	0.118883
FBB52	3,5-Diacetyltambulin	*TRPV1*	0.118883
FBB52	3,5-Diacetyltambulin	*TUBB1*	0.118883
FBB52	3,5-Diacetyltambulin	*XDH*	0.118883
FBB43	(3S)-5,7-dihydroxy-3-[(4-methoxyphenyl)methyl]-6-methyl-2,3-dihydrochromen-4-one*	*ADORA1*	0.118277
FBB43	(3S)-5,7-dihydroxy-3-[(4-methoxyphenyl)methyl]-6-methyl-2,3-dihydrochromen-4-one*	*CA12*	0.118277
FBB43	(3S)-5,7-dihydroxy-3-[(4-methoxyphenyl)methyl]-6-methyl-2,3-dihydrochromen-4-one*	*KDR*	0.118277
FBB66	Sexangularetin	*ABCB1*	0.118277
FBB66	Sexangularetin	*CA12*	0.118277
FBB66	Sexangularetin	*PFKFB3*	0.118277
FBB66	Sexangularetin	*PTPRS*	0.118277
FBB66	Sexangularetin	*XDH*	0.118277
FBB125	3-O-Acetylpinobanksin	*ABCB1*	0.118277
FBB125	3-O-Acetylpinobanksin	*MAPT*	0.118277
FBB125	3-O-Acetylpinobanksin	*MMP2*	0.118277
FBB157	Gossypetin	*APP*	0.118277
FBB65	3-O-Methylquercetin	*TERT*	0.118277
FBB23	5,7,5'-Trimethoxy-3',4'-methylenedioxyflavonoid	*ABCC1*	0.117455
FBB19	Thymonin	*ADORA1*	0.117455
FBB19	Thymonin	*CA12*	0.117455
FBB19	Thymonin	*CA4*	0.117455
FBB19	Thymonin	*PTGS2*	0.117455

ID	Compounds	gene	Probability
FBB46	Eupatorin-5-methylether	*CDK5*	0.117455
FBB46	Eupatorin-5-methylether	*FLT3*	0.117455
FBB46	Eupatorin-5-methylether	*PLG*	0.117455
FBB46	Eupatorin-5-methylether	*PTPRS*	0.117455
FBB161	Artemetin	*ABCB1*	0.115737
FBB161	Artemetin	*ABCC1*	0.115737
FBB161	Artemetin	*ACHE*	0.115737
FBB161	Artemetin	*AKR1A1*	0.115737
FBB161	Artemetin	*AKR1C2*	0.115737
FBB161	Artemetin	*AKT1*	0.115737
FBB161	Artemetin	*ALK*	0.115737
FBB161	Artemetin	*AXL*	0.115737
FBB161	Artemetin	*CA4*	0.115737
FBB161	Artemetin	*CA5A*	0.115737
FBB161	Artemetin	*CDK6*	0.115737
FBB161	Artemetin	*CXCR1*	0.115737
FBB161	Artemetin	*DAPK1*	0.115737
FBB161	Artemetin	*EGFR*	0.115737
FBB161	Artemetin	*F2*	0.115737
FBB161	Artemetin	*FLT3*	0.115737
FBB161	Artemetin	*GPR35*	0.115737
FBB161	Artemetin	*GSK3B*	0.115737
FBB161	Artemetin	*IGF1R*	0.115737
FBB161	Artemetin	*INSR*	0.115737
FBB161	Artemetin	*KDR*	0.115737
FBB161	Artemetin	*KIT*	0.115737
FBB161	Artemetin	*MAPT*	0.115737
FBB161	Artemetin	*MET*	0.115737
FBB161	Artemetin	*MMP2*	0.115737
FBB161	Artemetin	*MMP3*	0.115737
FBB161	Artemetin	*MMP9*	0.115737
FBB161	Artemetin	*MPO*	0.115737
FBB161	Artemetin	*MYLK*	0.115737
FBB161	Artemetin	*NOS2*	0.115737
FBB161	Artemetin	*OPRM1*	0.115737
FBB161	Artemetin	*PIK3R1*	0.115737
FBB161	Artemetin	*PIM1*	0.115737
FBB161	Artemetin	*PLA2G1B*	0.115737
FBB161	Artemetin	*PLA2G2A*	0.115737
FBB161	Artemetin	*PTGS2*	0.115737
FBB161	Artemetin	*PTK2*	0.115737
FBB161	Artemetin	*PTPRS*	0.115737
FBB161	Artemetin	*SLC22A12*	0.115737
FBB161	Artemetin	*SRC*	0.115737
FBB161	Artemetin	*ST6GAL1*	0.115737
FBB161	Artemetin	*TOP2A*	0.115737
FBB20	5,6,7,5'-tetramethoxy-3',4'-methylenedioxyflavonoid	*ABCB1*	0.115737
FBB20	5,6,7,5'-tetramethoxy-3',4'-methylenedioxyflavonoid	*ABCC1*	0.115737

ID	Compounds	gene	Probability
FBB20	5,6,7,5'-tetramethoxy-3',4'-methylenedioxyflavonoid	*ADORA1*	0.115737
FBB20	5,6,7,5'-tetramethoxy-3',4'-methylenedioxyflavonoid	*ADORA2A*	0.115737
FBB20	5,6,7,5'-tetramethoxy-3',4'-methylenedioxyflavonoid	*ADORA3*	0.115737
FBB20	5,6,7,5'-tetramethoxy-3',4'-methylenedioxyflavonoid	*AKR1A1*	0.115737
FBB20	5,6,7,5'-tetramethoxy-3',4'-methylenedioxyflavonoid	*AKR1C2*	0.115737
FBB20	5,6,7,5'-tetramethoxy-3',4'-methylenedioxyflavonoid	*ALOX5*	0.115737
FBB20	5,6,7,5'-tetramethoxy-3',4'-methylenedioxyflavonoid	*APP*	0.115737
FBB20	5,6,7,5'-tetramethoxy-3',4'-methylenedioxyflavonoid	*CA12*	0.115737
FBB20	5,6,7,5'-tetramethoxy-3',4'-methylenedioxyflavonoid	*CA4*	0.115737
FBB20	5,6,7,5'-tetramethoxy-3',4'-methylenedioxyflavonoid	*CDK5*	0.115737
FBB20	5,6,7,5'-tetramethoxy-3',4'-methylenedioxyflavonoid	*CDK6*	0.115737
FBB20	5,6,7,5'-tetramethoxy-3',4'-methylenedioxyflavonoid	*EGFR*	0.115737
FBB20	5,6,7,5'-tetramethoxy-3',4'-methylenedioxyflavonoid	*ESR2*	0.115737
FBB20	5,6,7,5'-tetramethoxy-3',4'-methylenedioxyflavonoid	*FLT3*	0.115737
FBB20	5,6,7,5'-tetramethoxy-3',4'-methylenedioxyflavonoid	*GPR35*	0.115737
FBB20	5,6,7,5'-tetramethoxy-3',4'-methylenedioxyflavonoid	*GRK6*	0.115737
FBB20	5,6,7,5'-tetramethoxy-3',4'-methylenedioxyflavonoid	*GSK3B*	0.115737
FBB20	5,6,7,5'-tetramethoxy-3',4'-methylenedioxyflavonoid	*KDR*	0.115737
FBB20	5,6,7,5'-tetramethoxy-3',4'-methylenedioxyflavonoid	*KIT*	0.115737
FBB20	5,6,7,5'-tetramethoxy-3',4'-methylenedioxyflavonoid	*LRRK2*	0.115737
FBB20	5,6,7,5'-tetramethoxy-3',4'-methylenedioxyflavonoid	*MAPK9*	0.115737
FBB20	5,6,7,5'-tetramethoxy-3',4'-methylenedioxyflavonoid	*MAPT*	0.115737
FBB20	5,6,7,5'-tetramethoxy-3',4'-methylenedioxyflavonoid	*MET*	0.115737
FBB20	5,6,7,5'-tetramethoxy-3',4'-methylenedioxyflavonoid	*MMP12*	0.115737
FBB20	5,6,7,5'-tetramethoxy-3',4'-methylenedioxyflavonoid	*MMP2*	0.115737
FBB20	5,6,7,5'-tetramethoxy-3',4'-methylenedioxyflavonoid	*MMP9*	0.115737
FBB20	5,6,7,5'-tetramethoxy-3',4'-methylenedioxyflavonoid	*NOS2*	0.115737
FBB20	5,6,7,5'-tetramethoxy-3',4'-methylenedioxyflavonoid	*NTRK1*	0.115737
FBB20	5,6,7,5'-tetramethoxy-3',4'-methylenedioxyflavonoid	*OPRM1*	0.115737
FBB20	5,6,7,5'-tetramethoxy-3',4'-methylenedioxyflavonoid	*PDGFRB*	0.115737
FBB20	5,6,7,5'-tetramethoxy-3',4'-methylenedioxyflavonoid	*PDGFRB*	0.115737
FBB20	5,6,7,5'-tetramethoxy-3',4'-methylenedioxyflavonoid	*PIK3R1*	0.115737
FBB20	5,6,7,5'-tetramethoxy-3',4'-methylenedioxyflavonoid	*PIM1*	0.115737
FBB20	5,6,7,5'-tetramethoxy-3',4'-methylenedioxyflavonoid	*PLA2G1B*	0.115737

ID	Compounds	gene	Probability
FBB20	5,6,7,5'-tetramethoxy-3',4'-methylenedioxyflavonoid	*PLA2G2A*	0.115737
FBB20	5,6,7,5'-tetramethoxy-3',4'-methylenedioxyflavonoid	*PLG*	0.115737
FBB20	5,6,7,5'-tetramethoxy-3',4'-methylenedioxyflavonoid	*PTGS2*	0.115737
FBB20	5,6,7,5'-tetramethoxy-3',4'-methylenedioxyflavonoid	*PTK2*	0.115737
FBB20	5,6,7,5'-tetramethoxy-3',4'-methylenedioxyflavonoid	*PTPRS*	0.115737
FBB20	5,6,7,5'-tetramethoxy-3',4'-methylenedioxyflavonoid	*SRC*	0.115737
FBB20	5,6,7,5'-tetramethoxy-3',4'-methylenedioxyflavonoid	*ST6GAL1*	0.115737
FBB20	5,6,7,5'-tetramethoxy-3',4'-methylenedioxyflavonoid	*TERT*	0.115737
FBB20	5,6,7,5'-tetramethoxy-3',4'-methylenedioxyflavonoid	*TOP1*	0.115737
FBB20	5,6,7,5'-tetramethoxy-3',4'-methylenedioxyflavonoid	*TOP2A*	0.115737
FBB20	5,6,7,5'-tetramethoxy-3',4'-methylenedioxyflavonoid	*TTR*	0.115737
FBB20	5,6,7,5'-tetramethoxy-3',4'-methylenedioxyflavonoid	*XDH*	0.115737
FBB2	Nigrolineaxanthone K	*PTPN1*	0.114495
FBB29	Mimulone	*RXRA*	0.114495
FBB11	Quercetin-3,3'-dimethyl ether	*ADORA1*	0.112748
FBB11	Quercetin-3,3'-dimethyl ether	*EGFR*	0.112748
FBB11	Quercetin-3,3'-dimethyl ether	*IGF1R*	0.112748
FBB16	Ranupetin	*CDK5*	0.112748
FBB41	Eucalyptin	*ACHE*	0.112748
FBB41	Eucalyptin	*ADORA1*	0.112748
FBB41	Eucalyptin	*ADORA3*	0.112748
FBB41	Eucalyptin	*ALOX5*	0.112748
FBB13	Chrysin	*ALK*	0.112042
FBB13	Chrysin	*AXL*	0.112042
FBB13	Chrysin	*CYP1A2*	0.112042
FBB13	Chrysin	*F2*	0.112042
FBB13	Chrysin	*GPR35*	0.112042
FBB13	Chrysin	*IGF1R*	0.112042
FBB13	Chrysin	*KDR*	0.112042
FBB13	Chrysin	*KIT*	0.112042
FBB13	Chrysin	*NOS2*	0.112042
FBB13	Chrysin	*PDE5A*	0.112042
FBB13	Chrysin	*PLA2G2A*	0.112042
FBB13	Chrysin	*PLA2G4A*	0.112042
FBB13	Chrysin	*PLG*	0.112042
FBB13	Chrysin	*PTPN1*	0.112042
FBB13	Chrysin	*SRC*	0.112042
FBB17	7,4'-Dihydroxydihydroflavonoids	*ACHE*	0.112042
FBB17	7,4'-Dihydroxydihydroflavonoids	*CA5A*	0.112042
FBB17	7,4'-Dihydroxydihydroflavonoids	*CA5B*	0.112042
FBB17	7,4'-Dihydroxydihydroflavonoids	*CTSB*	0.112042
FBB17	7,4'-Dihydroxydihydroflavonoids	*CYP3A4*	0.112042
FBB17	7,4'-Dihydroxydihydroflavonoids	*DYRK1A*	0.112042
FBB17	7,4'-Dihydroxydihydroflavonoids	*EDNRA*	0.112042

ID	Compounds	gene	Probability
FBB17	7,4'-Dihydroxydihydroflavonoids	*F3*	0.112042
FBB17	7,4'-Dihydroxydihydroflavonoids	*FGFR1*	0.112042
FBB17	7,4'-Dihydroxydihydroflavonoids	*GRM5*	0.112042
FBB17	7,4'-Dihydroxydihydroflavonoids	*GSK3B*	0.112042
FBB17	7,4'-Dihydroxydihydroflavonoids	*HDAC9*	0.112042
FBB17	7,4'-Dihydroxydihydroflavonoids	*HNF4A*	0.112042
FBB17	7,4'-Dihydroxydihydroflavonoids	*IGF1R*	0.112042
FBB17	7,4'-Dihydroxydihydroflavonoids	*INSR*	0.112042
FBB17	7,4'-Dihydroxydihydroflavonoids	*KDR*	0.112042
FBB17	7,4'-Dihydroxydihydroflavonoids	*KIT*	0.112042
FBB17	7,4'-Dihydroxydihydroflavonoids	*MET*	0.112042
FBB17	7,4'-Dihydroxydihydroflavonoids	*MMP2*	0.112042
FBB17	7,4'-Dihydroxydihydroflavonoids	*MMP3*	0.112042
FBB17	7,4'-Dihydroxydihydroflavonoids	*MMP9*	0.112042
FBB17	7,4'-Dihydroxydihydroflavonoids	*PIK3CA*	0.112042
FBB17	7,4'-Dihydroxydihydroflavonoids	*PLA2G10*	0.112042
FBB17	7,4'-Dihydroxydihydroflavonoids	*PLA2G2A*	0.112042
FBB17	7,4'-Dihydroxydihydroflavonoids	*PLA2G5*	0.112042
FBB17	7,4'-Dihydroxydihydroflavonoids	*PTGER1*	0.112042
FBB17	7,4'-Dihydroxydihydroflavonoids	*PTGER2*	0.112042
FBB17	7,4'-Dihydroxydihydroflavonoids	*PTGER3*	0.112042
FBB17	7,4'-Dihydroxydihydroflavonoids	*PTGER4*	0.112042
FBB17	7,4'-Dihydroxydihydroflavonoids	*PTPN1*	0.112042
FBB17	7,4'-Dihydroxydihydroflavonoids	*RXRA*	0.112042
FBB17	7,4'-Dihydroxydihydroflavonoids	*SERPINE1*	0.112042
FBB17	7,4'-Dihydroxydihydroflavonoids	*SIRT2*	0.112042
FBB17	7,4'-Dihydroxydihydroflavonoids	*SLC5A2*	0.112042
FBB17	7,4'-Dihydroxydihydroflavonoids	*SRC*	0.112042
FBB17	7,4'-Dihydroxydihydroflavonoids	*VCP*	0.112042
FBB17	7,4'-Dihydroxydihydroflavonoids	*VEGFA*	0.112042
FBB17	7,4'-Dihydroxydihydroflavonoids	*WEE1*	0.112042
FBB18	4'-Hydroxy-5,7-dimethoxyflavanone	*ABCB1*	0.111502
FBB18	4'-Hydroxy-5,7-dimethoxyflavanone	*ABL1*	0.111502
FBB18	4'-Hydroxy-5,7-dimethoxyflavanone	*ACHE*	0.111502
FBB18	4'-Hydroxy-5,7-dimethoxyflavanone	*ADORA2A*	0.111502
FBB18	4'-Hydroxy-5,7-dimethoxyflavanone	*ANPEP*	0.111502
FBB18	4'-Hydroxy-5,7-dimethoxyflavanone	*CA5A*	0.111502
FBB18	4'-Hydroxy-5,7-dimethoxyflavanone	*CA5B*	0.111502
FBB18	4'-Hydroxy-5,7-dimethoxyflavanone	*CCND1*	0.111502
FBB18	4'-Hydroxy-5,7-dimethoxyflavanone	*CCND1*	0.111502
FBB18	4'-Hydroxy-5,7-dimethoxyflavanone	*CSF1R*	0.111502
FBB18	4'-Hydroxy-5,7-dimethoxyflavanone	*CTSB*	0.111502
FBB18	4'-Hydroxy-5,7-dimethoxyflavanone	*DPP4*	0.111502

ID	Compounds	gene	Probability
FBB18	4'-Hydroxy-5,7-dimethoxyflavanone	*EDNRA*	0.111502
FBB18	4'-Hydroxy-5,7-dimethoxyflavanone	*EP300*	0.111502
FBB18	4'-Hydroxy-5,7-dimethoxyflavanone	*EZR*	0.111502
FBB18	4'-Hydroxy-5,7-dimethoxyflavanone	*FGFR1*	0.111502
FBB18	4'-Hydroxy-5,7-dimethoxyflavanone	*FLT4*	0.111502
FBB18	4'-Hydroxy-5,7-dimethoxyflavanone	*GRM5*	0.111502
FBB18	4'-Hydroxy-5,7-dimethoxyflavanone	*GSK3B*	0.111502
FBB18	4'-Hydroxy-5,7-dimethoxyflavanone	*HDAC9*	0.111502
FBB18	4'-Hydroxy-5,7-dimethoxyflavanone	*KDR*	0.111502
FBB18	4'-Hydroxy-5,7-dimethoxyflavanone	*KIT*	0.111502
FBB18	4'-Hydroxy-5,7-dimethoxyflavanone	*LRRK2*	0.111502
FBB18	4'-Hydroxy-5,7-dimethoxyflavanone	*MAP2K1*	0.111502
FBB18	4'-Hydroxy-5,7-dimethoxyflavanone	*MET*	0.111502
FBB18	4'-Hydroxy-5,7-dimethoxyflavanone	*MMP16*	0.111502
FBB18	4'-Hydroxy-5,7-dimethoxyflavanone	*MMP2*	0.111502
FBB18	4'-Hydroxy-5,7-dimethoxyflavanone	*MMP3*	0.111502
FBB18	4'-Hydroxy-5,7-dimethoxyflavanone	*MMP7*	0.111502
FBB18	4'-Hydroxy-5,7-dimethoxyflavanone	*MMP9*	0.111502
FBB18	4'-Hydroxy-5,7-dimethoxyflavanone	*MTOR*	0.111502
FBB18	4'-Hydroxy-5,7-dimethoxyflavanone	*HDAC3*	0.111502
FBB18	4'-Hydroxy-5,7-dimethoxyflavanone	*HDAC3*	0.111502
FBB18	4'-Hydroxy-5,7-dimethoxyflavanone	*PDGFRB*	0.111502
FBB18	4'-Hydroxy-5,7-dimethoxyflavanone	*PIK3C2G*	0.111502
FBB18	4'-Hydroxy-5,7-dimethoxyflavanone	*PIP4K2C*	0.111502
FBB18	4'-Hydroxy-5,7-dimethoxyflavanone	*PIP5K1C*	0.111502
FBB18	4'-Hydroxy-5,7-dimethoxyflavanone	*PLA2G10*	0.111502
FBB18	4'-Hydroxy-5,7-dimethoxyflavanone	*PLA2G2A*	0.111502
FBB18	4'-Hydroxy-5,7-dimethoxyflavanone	*PLA2G5*	0.111502
FBB18	4'-Hydroxy-5,7-dimethoxyflavanone	*SERPINE1*	0.111502
FBB18	4'-Hydroxy-5,7-dimethoxyflavanone	*SLC5A2*	0.111502
FBB18	4'-Hydroxy-5,7-dimethoxyflavanone	*SRC*	0.111502
FBB18	4'-Hydroxy-5,7-dimethoxyflavanone	*TAOK2*	0.111502
FBB18	4'-Hydroxy-5,7-dimethoxyflavanone	*WEE1*	0.111502
FBB9	2',7-Dihydroxy-3',4'-dimethoxyisoflavan	*ABL1*	0.111502

ID	Compounds	gene	Probability
FBB9	2',7-Dihydroxy-3',4'-dimethoxyisoflavan	*ADAM10*	0.111502
FBB9	2',7-Dihydroxy-3',4'-dimethoxyisoflavan	*ADAM17*	0.111502
FBB9	2',7-Dihydroxy-3',4'-dimethoxyisoflavan	*ADORA2A*	0.111502
FBB9	2',7-Dihydroxy-3',4'-dimethoxyisoflavan	*AKT1*	0.111502
FBB9	2',7-Dihydroxy-3',4'-dimethoxyisoflavan	*BRAF*	0.111502
FBB9	2',7-Dihydroxy-3',4'-dimethoxyisoflavan	*CA12*	0.111502
FBB9	2',7-Dihydroxy-3',4'-dimethoxyisoflavan	*CASP3*	0.111502
FBB9	2',7-Dihydroxy-3',4'-dimethoxyisoflavan	*CCND1*	0.111502
FBB9	2',7-Dihydroxy-3',4'-dimethoxyisoflavan	*CCND1*	0.111502
FBB9	2',7-Dihydroxy-3',4'-dimethoxyisoflavan	*CTSS*	0.111502
FBB9	2',7-Dihydroxy-3',4'-dimethoxyisoflavan	*ECE1*	0.111502
FBB9	2',7-Dihydroxy-3',4'-dimethoxyisoflavan	*EGFR*	0.111502
FBB9	2',7-Dihydroxy-3',4'-dimethoxyisoflavan	*EPHB2*	0.111502
FBB9	2',7-Dihydroxy-3',4'-dimethoxyisoflavan	*EZR*	0.111502
FBB9	2',7-Dihydroxy-3',4'-dimethoxyisoflavan	*FLT1*	0.111502
FBB9	2',7-Dihydroxy-3',4'-dimethoxyisoflavan	*GSK3B*	0.111502
FBB9	2',7-Dihydroxy-3',4'-dimethoxyisoflavan	*HDAC3*	0.111502
FBB9	2',7-Dihydroxy-3',4'-dimethoxyisoflavan	*HDAC9*	0.111502
FBB9	2',7-Dihydroxy-3',4'-dimethoxyisoflavan	*HTT*	0.111502
FBB9	2',7-Dihydroxy-3',4'-dimethoxyisoflavan	*KDR*	0.111502
FBB9	2',7-Dihydroxy-3',4'-dimethoxyisoflavan	*LCK*	0.111502
FBB9	2',7-Dihydroxy-3',4'-dimethoxyisoflavan	*MAPK1*	0.111502
FBB9	2',7-Dihydroxy-3',4'-dimethoxyisoflavan	*MAPKAPK2*	0.111502
FBB9	2',7-Dihydroxy-3',4'-dimethoxyisoflavan	*MET*	0.111502
FBB9	2',7-Dihydroxy-3',4'-dimethoxyisoflavan	*MIF*	0.111502
FBB9	2',7-Dihydroxy-3',4'-dimethoxyisoflavan	*MMP1*	0.111502
FBB9	2',7-Dihydroxy-3',4'-dimethoxyisoflavan	*MMP16*	0.111502
FBB9	2',7-Dihydroxy-3',4'-dimethoxyisoflavan	*MMP25*	0.111502
FBB9	2',7-Dihydroxy-3',4'-dimethoxyisoflavan	*MMP7*	0.111502
FBB9	2',7-Dihydroxy-3',4'-dimethoxyisoflavan	*MTOR*	0.111502
FBB9	2',7-Dihydroxy-3',4'-dimethoxyisoflavan	*HDAC3*	0.111502
FBB9	2',7-Dihydroxy-3',4'-dimethoxyisoflavan	*HDAC3*	0.111502
FBB9	2',7-Dihydroxy-3',4'-dimethoxyisoflavan	*NR4A1*	0.111502
FBB9	2',7-Dihydroxy-3',4'-dimethoxyisoflavan	*PDE10A*	0.111502

ID	Compounds	gene	Probability
FBB9	2',7-Dihydroxy-3',4'-dimethoxyisoflavan	*PIK3CA*	0.111502
FBB9	2',7-Dihydroxy-3',4'-dimethoxyisoflavan	*PIK3R1*	0.111502
FBB9	2',7-Dihydroxy-3',4'-dimethoxyisoflavan	*PIK3CD*	0.111502
FBB9	2',7-Dihydroxy-3',4'-dimethoxyisoflavan	*PIK3R1*	0.111502
FBB9	2',7-Dihydroxy-3',4'-dimethoxyisoflavan	*PIM1*	0.111502
FBB9	2',7-Dihydroxy-3',4'-dimethoxyisoflavan	*PTGS1*	0.111502
FBB9	2',7-Dihydroxy-3',4'-dimethoxyisoflavan	*ROCK1*	0.111502
FBB9	2',7-Dihydroxy-3',4'-dimethoxyisoflavan	*RPS6KA2*	0.111502
FBB9	2',7-Dihydroxy-3',4'-dimethoxyisoflavan	*SRC*	0.111502
FBB9	2',7-Dihydroxy-3',4'-dimethoxyisoflavan	*TAAR1*	0.111502
FBB9	2',7-Dihydroxy-3',4'-dimethoxyisoflavan	*TBK1*	0.111502
FBB9	2',7-Dihydroxy-3',4'-dimethoxyisoflavan	*TOP1*	0.111502
FBB9	2',7-Dihydroxy-3',4'-dimethoxyisoflavan	*VCP*	0.111502
FBB9	2',7-Dihydroxy-3',4'-dimethoxyisoflavan	*WEE1*	0.111502
FBB102	3-Methylkaempferol	*CFTR*	0.111502
FBB102	3-Methylkaempferol	*MAPT*	0.111502
FBB102	3-Methylkaempferol	*OPRM1*	0.111502
FBB102	3-Methylkaempferol	*TERT*	0.111502
FBB102	3-Methylkaempferol	*TTR*	0.111502
FBB123	Morin	*AR*	0.111502
FBB123	Morin	*EGFR*	0.111502
FBB123	Morin	*GSK3B*	0.111502
FBB123	Morin	*IGF1R*	0.111502
FBB123	Morin	*INSR*	0.111502
FBB123	Morin	*KDR*	0.111502
FBB123	Morin	*PTK2*	0.111502
FBB123	Morin	*SRC*	0.111502
FBB123	Morin	*TERT*	0.111502
FBB43	(3S)-5,7-dihydroxy-3-[(4-methoxyphenyl)methyl]-6-methyl-2,3-dihydrochromen-4-one*	*ABCC1*	0.109946
FBB43	(3S)-5,7-dihydroxy-3-[(4-methoxyphenyl)methyl]-6-methyl-2,3-dihydrochromen-4-one*	*ADORA3*	0.109946
FBB43	(3S)-5,7-dihydroxy-3-[(4-methoxyphenyl)methyl]-6-methyl-2,3-dihydrochromen-4-one*	*ESR2*	0.109946
FBB43	(3S)-5,7-dihydroxy-3-[(4-methoxyphenyl)methyl]-6-methyl-2,3-dihydrochromen-4-one*	*MET*	0.109946
FBB43	(3S)-5,7-dihydroxy-3-[(4-methoxyphenyl)methyl]-6-methyl-2,3-dihydrochromen-4-one*	*PTGS1*	0.109946
FBB66	Sexangularetin	*ABCC1*	0.109946
FBB66	Sexangularetin	*ALOX5*	0.109946
FBB66	Sexangularetin	*CA4*	0.109946
FBB66	Sexangularetin	*FLT3*	0.109946

ID	Compounds	gene	Probability
FBB66	Sexangularetin	*IGF1R*	0.109946
FBB66	Sexangularetin	*KIT*	0.109946
FBB66	Sexangularetin	*OPRM1*	0.109946
FBB66	Sexangularetin	*SLC22A12*	0.109946
FBB66	Sexangularetin	*TERT*	0.109946
FBB125	3-O-Acetylpinobanksin	*APP*	0.109946
FBB125	3-O-Acetylpinobanksin	*CA12*	0.109946
FBB125	3-O-Acetylpinobanksin	*CA4*	0.109946
FBB125	3-O-Acetylpinobanksin	*DYRK1A*	0.109946
FBB125	3-O-Acetylpinobanksin	*MMP12*	0.109946
FBB125	3-O-Acetylpinobanksin	*TERT*	0.109946
FBB157	Gossypetin	*GRK6*	0.109946
FBB157	Gossypetin	*TTR*	0.109946
FBB65	3-O-Methylquercetin	*CD38*	0.109946
FBB65	3-O-Methylquercetin	*ESR2*	0.109946
FBB65	3-O-Methylquercetin	*MMP12*	0.109946
FBB65	3-O-Methylquercetin	*PTPRS*	0.109946
FBB65	3-O-Methylquercetin	*SLC22A12*	0.109946
FBB65	3-O-Methylquercetin	*TTR*	0.109946
FBB23	5,7,5'-Trimethoxy-3',4'-methylenedioxyflavonoid	*ABCB1*	0.10934
FBB23	5,7,5'-Trimethoxy-3',4'-methylenedioxyflavonoid	*ACHE*	0.10934
FBB23	5,7,5'-Trimethoxy-3',4'-methylenedioxyflavonoid	*ADORA1*	0.10934
FBB23	5,7,5'-Trimethoxy-3',4'-methylenedioxyflavonoid	*ADORA2A*	0.10934
FBB23	5,7,5'-Trimethoxy-3',4'-methylenedioxyflavonoid	*ADORA3*	0.10934
FBB23	5,7,5'-Trimethoxy-3',4'-methylenedioxyflavonoid	*AKR1C2*	0.10934
FBB23	5,7,5'-Trimethoxy-3',4'-methylenedioxyflavonoid	*ALK*	0.10934
FBB23	5,7,5'-Trimethoxy-3',4'-methylenedioxyflavonoid	*ALOX5*	0.10934
FBB23	5,7,5'-Trimethoxy-3',4'-methylenedioxyflavonoid	*APP*	0.10934
FBB23	5,7,5'-Trimethoxy-3',4'-methylenedioxyflavonoid	*AXL*	0.10934
FBB23	5,7,5'-Trimethoxy-3',4'-methylenedioxyflavonoid	*CA12*	0.10934
FBB23	5,7,5'-Trimethoxy-3',4'-methylenedioxyflavonoid	*CA4*	0.10934
FBB23	5,7,5'-Trimethoxy-3',4'-methylenedioxyflavonoid	*CA5A*	0.10934
FBB23	5,7,5'-Trimethoxy-3',4'-methylenedioxyflavonoid	*CD38*	0.10934
FBB23	5,7,5'-Trimethoxy-3',4'-methylenedioxyflavonoid	*CDK5*	0.10934
FBB23	5,7,5'-Trimethoxy-3',4'-methylenedioxyflavonoid	*CFTR*	0.10934
FBB23	5,7,5'-Trimethoxy-3',4'-methylenedioxyflavonoid	*DAPK1*	0.10934
FBB23	5,7,5'-Trimethoxy-3',4'-methylenedioxyflavonoid	*EGFR*	0.10934
FBB23	5,7,5'-Trimethoxy-3',4'-methylenedioxyflavonoid	*EGLN1*	0.10934
FBB23	5,7,5'-Trimethoxy-3',4'-methylenedioxyflavonoid	*ESR1*	0.10934
FBB23	5,7,5'-Trimethoxy-3',4'-methylenedioxyflavonoid	*ESR2*	0.10934

ID	Compounds	gene	Probability
FBB23	5,7,5'-Trimethoxy-3',4'-methylenedioxyflavonoid	*F2*	0.10934
FBB23	5,7,5'-Trimethoxy-3',4'-methylenedioxyflavonoid	*FLT3*	0.10934
FBB23	5,7,5'-Trimethoxy-3',4'-methylenedioxyflavonoid	*GSK3B*	0.10934
FBB23	5,7,5'-Trimethoxy-3',4'-methylenedioxyflavonoid	*IGF1R*	0.10934
FBB23	5,7,5'-Trimethoxy-3',4'-methylenedioxyflavonoid	*INSR*	0.10934
FBB23	5,7,5'-Trimethoxy-3',4'-methylenedioxyflavonoid	*KDR*	0.10934
FBB23	5,7,5'-Trimethoxy-3',4'-methylenedioxyflavonoid	*KIT*	0.10934
FBB23	5,7,5'-Trimethoxy-3',4'-methylenedioxyflavonoid	*MAPK1*	0.10934
FBB23	5,7,5'-Trimethoxy-3',4'-methylenedioxyflavonoid	*MAPK14*	0.10934
FBB23	5,7,5'-Trimethoxy-3',4'-methylenedioxyflavonoid	*MAPK9*	0.10934
FBB23	5,7,5'-Trimethoxy-3',4'-methylenedioxyflavonoid	*MET*	0.10934
FBB23	5,7,5'-Trimethoxy-3',4'-methylenedioxyflavonoid	*MMP12*	0.10934
FBB23	5,7,5'-Trimethoxy-3',4'-methylenedioxyflavonoid	*MMP2*	0.10934
FBB23	5,7,5'-Trimethoxy-3',4'-methylenedioxyflavonoid	*MMP9*	0.10934
FBB23	5,7,5'-Trimethoxy-3',4'-methylenedioxyflavonoid	*MTOR*	0.10934
FBB23	5,7,5'-Trimethoxy-3',4'-methylenedioxyflavonoid	*MYLK*	0.10934
FBB23	5,7,5'-Trimethoxy-3',4'-methylenedioxyflavonoid	*NTRK1*	0.10934
FBB23	5,7,5'-Trimethoxy-3',4'-methylenedioxyflavonoid	*OPRM1*	0.10934
FBB23	5,7,5'-Trimethoxy-3',4'-methylenedioxyflavonoid	*PIK3CA*	0.10934
FBB23	5,7,5'-Trimethoxy-3',4'-methylenedioxyflavonoid	*PIM1*	0.10934
FBB23	5,7,5'-Trimethoxy-3',4'-methylenedioxyflavonoid	*PLA2G1B*	0.10934
FBB23	5,7,5'-Trimethoxy-3',4'-methylenedioxyflavonoid	*PLA2G2A*	0.10934
FBB23	5,7,5'-Trimethoxy-3',4'-methylenedioxyflavonoid	*PLG*	0.10934
FBB23	5,7,5'-Trimethoxy-3',4'-methylenedioxyflavonoid	*PTGS2*	0.10934
FBB23	5,7,5'-Trimethoxy-3',4'-methylenedioxyflavonoid	*PTK2*	0.10934
FBB23	5,7,5'-Trimethoxy-3',4'-methylenedioxyflavonoid	*PTPRS*	0.10934
FBB23	5,7,5'-Trimethoxy-3',4'-methylenedioxyflavonoid	*SIRT2*	0.10934
FBB23	5,7,5'-Trimethoxy-3',4'-methylenedioxyflavonoid	*SRC*	0.10934
FBB23	5,7,5'-Trimethoxy-3',4'-methylenedioxyflavonoid	*ST6GAL1*	0.10934
FBB23	5,7,5'-Trimethoxy-3',4'-methylenedioxyflavonoid	*TERT*	0.10934
FBB23	5,7,5'-Trimethoxy-3',4'-methylenedioxyflavonoid	*TOP2A*	0.10934
FBB23	5,7,5'-Trimethoxy-3',4'-methylenedioxyflavonoid	*XDH*	0.10934
FBB19	Thymonin	*ABCB1*	0.10934
FBB19	Thymonin	*ABCC1*	0.10934
FBB19	Thymonin	*ACHE*	0.10934

ID	Compounds	gene	Probability
FBB19	Thymonin	*ADORA2A*	0.10934
FBB19	Thymonin	*ADORA3*	0.10934
FBB19	Thymonin	*AKR1A1*	0.10934
FBB19	Thymonin	*AKR1C2*	0.10934
FBB19	Thymonin	*APP*	0.10934
FBB19	Thymonin	*CA5A*	0.10934
FBB19	Thymonin	*CD38*	0.10934
FBB19	Thymonin	*CDK5*	0.10934
FBB19	Thymonin	*CFTR*	0.10934
FBB19	Thymonin	*CYP1A1*	0.10934
FBB19	Thymonin	*CYP1A2*	0.10934
FBB19	Thymonin	*DAPK1*	0.10934
FBB19	Thymonin	*EGFR*	0.10934
FBB19	Thymonin	*ESR1*	0.10934
FBB19	Thymonin	*ESR2*	0.10934
FBB19	Thymonin	*F2*	0.10934
FBB19	Thymonin	*FLT3*	0.10934
FBB19	Thymonin	*GPR35*	0.10934
FBB19	Thymonin	*GSK3B*	0.10934
FBB19	Thymonin	*KDR*	0.10934
FBB19	Thymonin	*KIT*	0.10934
FBB19	Thymonin	*MET*	0.10934
FBB19	Thymonin	*MMP12*	0.10934
FBB19	Thymonin	*MMP2*	0.10934
FBB19	Thymonin	*MMP3*	0.10934
FBB19	Thymonin	*MMP9*	0.10934
FBB19	Thymonin	*MYLK*	0.10934
FBB19	Thymonin	*NOS2*	0.10934
FBB19	Thymonin	*PFKFB3*	0.10934
FBB19	Thymonin	*PIM1*	0.10934
FBB19	Thymonin	*PLA2G1B*	0.10934
FBB19	Thymonin	*PLA2G2A*	0.10934
FBB19	Thymonin	*PLG*	0.10934
FBB19	Thymonin	*PTK2*	0.10934
FBB19	Thymonin	*PTPRS*	0.10934
FBB19	Thymonin	*SLC22A12*	0.10934
FBB19	Thymonin	*SRC*	0.10934
FBB19	Thymonin	*TOP1*	0.10934
FBB19	Thymonin	*TOP2A*	0.10934
FBB46	Eupatorin-5-methylether	*ABCB1*	0.10934
FBB46	Eupatorin-5-methylether	*ACHE*	0.10934
FBB46	Eupatorin-5-methylether	*AKR1A1*	0.10934
FBB46	Eupatorin-5-methylether	*ALK*	0.10934
FBB46	Eupatorin-5-methylether	*BCL2*	0.10934
FBB46	Eupatorin-5-methylether	*CA12*	0.10934
FBB46	Eupatorin-5-methylether	*CA4*	0.10934
FBB46	Eupatorin-5-methylether	*CA5A*	0.10934
FBB46	Eupatorin-5-methylether	*CD38*	0.10934
FBB46	Eupatorin-5-methylether	*EGFR*	0.10934

ID	Compounds	gene	Probability
FBB46	Eupatorin-5-methylether	*ESR1*	0.10934
FBB46	Eupatorin-5-methylether	*ESR2*	0.10934
FBB46	Eupatorin-5-methylether	*GPR35*	0.10934
FBB46	Eupatorin-5-methylether	*GRK6*	0.10934
FBB46	Eupatorin-5-methylether	*KDR*	0.10934
FBB46	Eupatorin-5-methylether	*MET*	0.10934
FBB46	Eupatorin-5-methylether	*MMP12*	0.10934
FBB46	Eupatorin-5-methylether	*MMP3*	0.10934
FBB46	Eupatorin-5-methylether	*MPO*	0.10934
FBB46	Eupatorin-5-methylether	*NOS2*	0.10934
FBB46	Eupatorin-5-methylether	*OPRM1*	0.10934
FBB46	Eupatorin-5-methylether	*PFKFB3*	0.10934
FBB46	Eupatorin-5-methylether	*PIK3R1*	0.10934
FBB46	Eupatorin-5-methylether	*PLA2G1B*	0.10934
FBB46	Eupatorin-5-methylether	*PLA2G2A*	0.10934
FBB46	Eupatorin-5-methylether	*PTGS2*	0.10934
FBB46	Eupatorin-5-methylether	*PTK2*	0.10934
FBB46	Eupatorin-5-methylether	*SRC*	0.10934
FBB46	Eupatorin-5-methylether	*ST6GAL1*	0.10934
FBB46	Eupatorin-5-methylether	*TERT*	0.10934
FBB46	Eupatorin-5-methylether	*TOP1*	0.10934
FBB46	Eupatorin-5-methylether	*TOP2A*	0.10934
FBB46	Eupatorin-5-methylether	*TTR*	0.10934
FBB46	Eupatorin-5-methylether	*TUBB1*	0.10934
FBB46	Eupatorin-5-methylether	*XDH*	0.10934
FBB36	3'-Hydroxy-4'-O-methylglabridin	*ABL1*	0.10934
FBB36	3'-Hydroxy-4'-O-methylglabridin	*ADORA1*	0.10934
FBB36	3'-Hydroxy-4'-O-methylglabridin	*ADORA2A*	0.10934
FBB36	3'-Hydroxy-4'-O-methylglabridin	*ADORA3*	0.10934
FBB36	3'-Hydroxy-4'-O-methylglabridin	*ATR*	0.10934
FBB36	3'-Hydroxy-4'-O-methylglabridin	*BRAF*	0.10934
FBB36	3'-Hydroxy-4'-O-methylglabridin	*CDC25A*	0.10934
FBB36	3'-Hydroxy-4'-O-methylglabridin	*CRHR1*	0.10934
FBB36	3'-Hydroxy-4'-O-methylglabridin	*DRD3*	0.10934
FBB36	3'-Hydroxy-4'-O-methylglabridin	*EGLN1*	0.10934
FBB36	3'-Hydroxy-4'-O-methylglabridin	*ELANE*	0.10934
FBB36	3'-Hydroxy-4'-O-methylglabridin	*EPHB2*	0.10934
FBB36	3'-Hydroxy-4'-O-methylglabridin	*ESR1*	0.10934
FBB36	3'-Hydroxy-4'-O-methylglabridin	*ESR2*	0.10934
FBB36	3'-Hydroxy-4'-O-methylglabridin	*FGFR1*	0.10934
FBB36	3'-Hydroxy-4'-O-methylglabridin	*FLT3*	0.10934
FBB36	3'-Hydroxy-4'-O-methylglabridin	*GABRB2*	0.10934
FBB36	3'-Hydroxy-4'-O-methylglabridin	*GABRB2*	0.10934
FBB36	3'-Hydroxy-4'-O-methylglabridin	*HCK*	0.10934
FBB36	3'-Hydroxy-4'-O-methylglabridin	*HDAC1*	0.10934
FBB36	3'-Hydroxy-4'-O-methylglabridin	*HDAC8*	0.10934
FBB36	3'-Hydroxy-4'-O-methylglabridin	*HSD11B1*	0.10934
FBB36	3'-Hydroxy-4'-O-methylglabridin	*HTR1A*	0.10934
FBB36	3'-Hydroxy-4'-O-methylglabridin	*KDR*	0.10934

ID	Compounds	gene	Probability
FBB36	3'-Hydroxy-4'-O-methylglabridin	*KIT*	0.10934
FBB36	3'-Hydroxy-4'-O-methylglabridin	*LCK*	0.10934
FBB36	3'-Hydroxy-4'-O-methylglabridin	*MET*	0.10934
FBB36	3'-Hydroxy-4'-O-methylglabridin	*MPEG1*	0.10934
FBB36	3'-Hydroxy-4'-O-methylglabridin	*MTOR*	0.10934
FBB36	3'-Hydroxy-4'-O-methylglabridin	*MYLK*	0.10934
FBB36	3'-Hydroxy-4'-O-methylglabridin	*NOX1*	0.10934
FBB36	3'-Hydroxy-4'-O-methylglabridin	*PDE5A*	0.10934
FBB36	3'-Hydroxy-4'-O-methylglabridin	*PIK3CD*	0.10934
FBB36	3'-Hydroxy-4'-O-methylglabridin	*PIK3R1*	0.10934
FBB36	3'-Hydroxy-4'-O-methylglabridin	*PIM1*	0.10934
FBB36	3'-Hydroxy-4'-O-methylglabridin	*PLAA*	0.10934
FBB36	3'-Hydroxy-4'-O-methylglabridin	*PPIA*	0.10934
FBB36	3'-Hydroxy-4'-O-methylglabridin	*PTK2*	0.10934
FBB36	3'-Hydroxy-4'-O-methylglabridin	*PTPN1*	0.10934
FBB36	3'-Hydroxy-4'-O-methylglabridin	*ROCK1*	0.10934
FBB36	3'-Hydroxy-4'-O-methylglabridin	*SRC*	0.10934
FBB36	3'-Hydroxy-4'-O-methylglabridin	*TBXA2R*	0.10934
FBB36	3'-Hydroxy-4'-O-methylglabridin	*TERT*	0.10934
FBB36	3'-Hydroxy-4'-O-methylglabridin	*TGFBR2*	0.10934
FBB36	3'-Hydroxy-4'-O-methylglabridin	*THRA*	0.10934
FBB36	3'-Hydroxy-4'-O-methylglabridin	*TRPM8*	0.10934
FBB36	3'-Hydroxy-4'-O-methylglabridin	*TRPV1*	0.10934
FBB36	3'-Hydroxy-4'-O-methylglabridin	*VCP*	0.10934
FBB36	3'-Hydroxy-4'-O-methylglabridin	*WEE1*	0.10934
FBB38	7-Hydroxyflavone	*ABCC1*	0.108018
FBB38	7-Hydroxyflavone	*ALOX5*	0.108018
FBB38	7-Hydroxyflavone	*PTPRS*	0.108018
FBB2	Nigrolineaxanthone K	*ABL1*	0.106166
FBB2	Nigrolineaxanthone K	*ADAM17*	0.106166
FBB2	Nigrolineaxanthone K	*ADORA2A*	0.106166
FBB2	Nigrolineaxanthone K	*ADRA1A*	0.106166
FBB2	Nigrolineaxanthone K	*BRAF*	0.106166
FBB2	Nigrolineaxanthone K	*CA12*	0.106166
FBB2	Nigrolineaxanthone K	*CCND1*	0.106166
FBB2	Nigrolineaxanthone K	*CRHR1*	0.106166
FBB2	Nigrolineaxanthone K	*CSF1R*	0.106166
FBB2	Nigrolineaxanthone K	*CXCR1*	0.106166
FBB2	Nigrolineaxanthone K	*CXCR2*	0.106166
FBB2	Nigrolineaxanthone K	*DAPK1*	0.106166
FBB2	Nigrolineaxanthone K	*EGFR*	0.106166
FBB2	Nigrolineaxanthone K	*EGLN1*	0.106166
FBB2	Nigrolineaxanthone K	*ELANE*	0.106166
FBB2	Nigrolineaxanthone K	*EZR*	0.106166
FBB2	Nigrolineaxanthone K	*FLT1*	0.106166
FBB2	Nigrolineaxanthone K	*FLT3*	0.106166
FBB2	Nigrolineaxanthone K	*GRM5*	0.106166
FBB2	Nigrolineaxanthone K	*GSK3B*	0.106166
FBB2	Nigrolineaxanthone K	*HCK*	0.106166

ID	Compounds	gene	Probability
FBB2	Nigrolineaxanthone K	*HSD11B1*	0.106166
FBB2	Nigrolineaxanthone K	*LCK*	0.106166
FBB2	Nigrolineaxanthone K	*LIPG*	0.106166
FBB2	Nigrolineaxanthone K	*MAP2K1*	0.106166
FBB2	Nigrolineaxanthone K	*MAPK1*	0.106166
FBB2	Nigrolineaxanthone K	*MAPK10*	0.106166
FBB2	Nigrolineaxanthone K	*MAPK14*	0.106166
FBB2	Nigrolineaxanthone K	*MAPK8*	0.106166
FBB2	Nigrolineaxanthone K	*MAPK9*	0.106166
FBB2	Nigrolineaxanthone K	*MMP2*	0.106166
FBB2	Nigrolineaxanthone K	*MMP3*	0.106166
FBB2	Nigrolineaxanthone K	*MMP9*	0.106166
FBB2	Nigrolineaxanthone K	*MTOR*	0.106166
FBB2	Nigrolineaxanthone K	*NR4A1*	0.106166
FBB2	Nigrolineaxanthone K	*PDE10A*	0.106166
FBB2	Nigrolineaxanthone K	*PDGFRB*	0.106166
FBB2	Nigrolineaxanthone K	*PIK3CA*	0.106166
FBB2	Nigrolineaxanthone K	*PIK3CD*	0.106166
FBB2	Nigrolineaxanthone K	*PIM1*	0.106166
FBB2	Nigrolineaxanthone K	*PLA2G7*	0.106166
FBB2	Nigrolineaxanthone K	*PTGER2*	0.106166
FBB2	Nigrolineaxanthone K	*PTK2*	0.106166
FBB2	Nigrolineaxanthone K	*ROCK1*	0.106166
FBB2	Nigrolineaxanthone K	*RPS6KB1*	0.106166
FBB2	Nigrolineaxanthone K	*SPHK1*	0.106166
FBB2	Nigrolineaxanthone K	*SPHK2*	0.106166
FBB2	Nigrolineaxanthone K	*STAT3*	0.106166
FBB2	Nigrolineaxanthone K	*TBXA2R*	0.106166
FBB2	Nigrolineaxanthone K	*TERT*	0.106166
FBB2	Nigrolineaxanthone K	*TUBB1*	0.106166
FBB2	Nigrolineaxanthone K	*WEE1*	0.106166
FBB29	Mimulone	*ABCC1*	0.106166
FBB29	Mimulone	*ABL1*	0.106166
FBB29	Mimulone	*ADAM17*	0.106166
FBB29	Mimulone	*ADORA1*	0.106166
FBB29	Mimulone	*ADORA3*	0.106166
FBB29	Mimulone	*ALDH2*	0.106166
FBB29	Mimulone	*CA12*	0.106166
FBB29	Mimulone	*CA4*	0.106166
FBB29	Mimulone	*CASP1*	0.106166
FBB29	Mimulone	*CASP3*	0.106166
FBB29	Mimulone	*CDC25A*	0.106166
FBB29	Mimulone	*CPT1A*	0.106166
FBB29	Mimulone	*CTSK*	0.106166
FBB29	Mimulone	*F2*	0.106166
FBB29	Mimulone	*FAAH*	0.106166
FBB29	Mimulone	*GNRHR*	0.106166
FBB29	Mimulone	*GRM5*	0.106166
FBB29	Mimulone	*HDAC3*	0.106166

ID	Compounds	gene	Probability
FBB29	Mimulone	*LCK*	0.106166
FBB29	Mimulone	*LDLR*	0.106166
FBB29	Mimulone	*MGLL*	0.106166
FBB29	Mimulone	*MMP12*	0.106166
FBB29	Mimulone	*MMP25*	0.106166
FBB29	Mimulone	*MMP3*	0.106166
FBB29	Mimulone	*MPEG1*	0.106166
FBB29	Mimulone	*HDAC3*	0.106166
FBB29	Mimulone	*PDGFRB*	0.106166
FBB29	Mimulone	*PLA2G10*	0.106166
FBB29	Mimulone	*PLA2G5*	0.106166
FBB29	Mimulone	*PTGS1*	0.106166
FBB29	Mimulone	*PTK2B*	0.106166
FBB29	Mimulone	*PTPN1*	0.106166
FBB29	Mimulone	*S1PR1*	0.106166
FBB29	Mimulone	*SCN9A*	0.106166
FBB29	Mimulone	*SERPINE1*	0.106166
FBB29	Mimulone	*SLC5A2*	0.106166
FBB29	Mimulone	*SMO*	0.106166
FBB29	Mimulone	*TYMS*	0.106166
FBB29	Mimulone	*TYRO3*	0.106166
FBB11	Quercetin-3,3'-dimethyl ether	*ACHE*	0.104672
FBB11	Quercetin-3,3'-dimethyl ether	*ADORA2A*	0.104672
FBB11	Quercetin-3,3'-dimethyl ether	*ADORA3*	0.104672
FBB11	Quercetin-3,3'-dimethyl ether	*AKR1A1*	0.104672
FBB11	Quercetin-3,3'-dimethyl ether	*AKR1C2*	0.104672
FBB11	Quercetin-3,3'-dimethyl ether	*AKT1*	0.104672
FBB11	Quercetin-3,3'-dimethyl ether	*ALK*	0.104672
FBB11	Quercetin-3,3'-dimethyl ether	*ALOX5*	0.104672
FBB11	Quercetin-3,3'-dimethyl ether	*AXL*	0.104672
FBB11	Quercetin-3,3'-dimethyl ether	*CA5A*	0.104672
FBB11	Quercetin-3,3'-dimethyl ether	*CDK5*	0.104672
FBB11	Quercetin-3,3'-dimethyl ether	*CDK6*	0.104672
FBB11	Quercetin-3,3'-dimethyl ether	*CXCR1*	0.104672
FBB11	Quercetin-3,3'-dimethyl ether	*DAPK1*	0.104672
FBB11	Quercetin-3,3'-dimethyl ether	*ESR1*	0.104672
FBB11	Quercetin-3,3'-dimethyl ether	*ESR2*	0.104672
FBB11	Quercetin-3,3'-dimethyl ether	*FLT3*	0.104672
FBB11	Quercetin-3,3'-dimethyl ether	*GPR35*	0.104672
FBB11	Quercetin-3,3'-dimethyl ether	*GSK3B*	0.104672
FBB11	Quercetin-3,3'-dimethyl ether	*INSR*	0.104672
FBB11	Quercetin-3,3'-dimethyl ether	*KDR*	0.104672
FBB11	Quercetin-3,3'-dimethyl ether	*KIT*	0.104672
FBB11	Quercetin-3,3'-dimethyl ether	*MAPT*	0.104672
FBB11	Quercetin-3,3'-dimethyl ether	*MET*	0.104672
FBB11	Quercetin-3,3'-dimethyl ether	*MMP2*	0.104672
FBB11	Quercetin-3,3'-dimethyl ether	*MMP3*	0.104672
FBB11	Quercetin-3,3'-dimethyl ether	*MMP9*	0.104672
FBB11	Quercetin-3,3'-dimethyl ether	*MPO*	0.104672

ID	Compounds	gene	Probability
FBB11	Quercetin-3,3'-dimethyl ether	*MYLK*	0.104672
FBB11	Quercetin-3,3'-dimethyl ether	*NOS2*	0.104672
FBB11	Quercetin-3,3'-dimethyl ether	*OPRM1*	0.104672
FBB11	Quercetin-3,3'-dimethyl ether	*PIK3R1*	0.104672
FBB11	Quercetin-3,3'-dimethyl ether	*PIM1*	0.104672
FBB11	Quercetin-3,3'-dimethyl ether	*PLA2G1B*	0.104672
FBB11	Quercetin-3,3'-dimethyl ether	*PLA2G2A*	0.104672
FBB11	Quercetin-3,3'-dimethyl ether	*PTK2*	0.104672
FBB11	Quercetin-3,3'-dimethyl ether	*SLC22A12*	0.104672
FBB11	Quercetin-3,3'-dimethyl ether	*SRC*	0.104672
FBB11	Quercetin-3,3'-dimethyl ether	*ST6GAL1*	0.104672
FBB11	Quercetin-3,3'-dimethyl ether	*TERT*	0.104672
FBB11	Quercetin-3,3'-dimethyl ether	*TOP2A*	0.104672
FBB16	Ranupetin	*CDK6*	0.104672
FBB16	Ranupetin	*ESR2*	0.104672
FBB16	Ranupetin	*KIT*	0.104672
FBB16	Ranupetin	*NOS2*	0.104672
FBB16	Ranupetin	*PFKFB3*	0.104672
FBB16	Ranupetin	*PLG*	0.104672
FBB16	Ranupetin	*PTPRS*	0.104672
FBB16	Ranupetin	*SLC22A12*	0.104672
FBB16	Ranupetin	*ST6GAL1*	0.104672
FBB41	Eucalyptin	*ABCC1*	0.104672
FBB41	Eucalyptin	*ADORA2A*	0.104672
FBB41	Eucalyptin	*AKR1A1*	0.104672
FBB41	Eucalyptin	*AKR1C2*	0.104672
FBB41	Eucalyptin	*APP*	0.104672
FBB41	Eucalyptin	*AR*	0.104672
FBB41	Eucalyptin	*CA12*	0.104672
FBB41	Eucalyptin	*CA4*	0.104672
FBB41	Eucalyptin	*CA5A*	0.104672
FBB41	Eucalyptin	*CDK5*	0.104672
FBB41	Eucalyptin	*CDK6*	0.104672
FBB41	Eucalyptin	*CFTR*	0.104672
FBB41	Eucalyptin	*DRD3*	0.104672
FBB41	Eucalyptin	*ESR1*	0.104672
FBB41	Eucalyptin	*ESR2*	0.104672
FBB41	Eucalyptin	*F2*	0.104672
FBB41	Eucalyptin	*FLT3*	0.104672
FBB41	Eucalyptin	*GABRB2*	0.104672
FBB41	Eucalyptin	*GPR35*	0.104672
FBB41	Eucalyptin	*GRK6*	0.104672
FBB41	Eucalyptin	*GSK3B*	0.104672
FBB41	Eucalyptin	*KIT*	0.104672
FBB41	Eucalyptin	*LCK*	0.104672
FBB41	Eucalyptin	*MET*	0.104672
FBB41	Eucalyptin	*MMP12*	0.104672
FBB41	Eucalyptin	*MMP2*	0.104672
FBB41	Eucalyptin	*MMP3*	0.104672

ID	Compounds	gene	Probability
FBB41	Eucalyptin	*MMP9*	0.104672
FBB41	Eucalyptin	*NOS2*	0.104672
FBB41	Eucalyptin	*OPRM1*	0.104672
FBB41	Eucalyptin	*PDE5A*	0.104672
FBB41	Eucalyptin	*PIM1*	0.104672
FBB41	Eucalyptin	*PLA2G2A*	0.104672
FBB41	Eucalyptin	*PLG*	0.104672
FBB41	Eucalyptin	*PTGS2*	0.104672
FBB41	Eucalyptin	*PTPN1*	0.104672
FBB41	Eucalyptin	*PTPRS*	0.104672
FBB41	Eucalyptin	*SLC22A12*	0.104672
FBB41	Eucalyptin	*TERT*	0.104672
FBB41	Eucalyptin	*TTR*	0.104672
FBB41	Eucalyptin	*VEGFA*	0.104672
FBB41	Eucalyptin	*XDH*	0.104672
FBB151	Isobavachin	*ABCC1*	0.104672
FBB151	Isobavachin	*ABL1*	0.104672
FBB151	Isobavachin	*ADAM17*	0.104672
FBB151	Isobavachin	*ADORA1*	0.104672
FBB151	Isobavachin	*ADORA3*	0.104672
FBB151	Isobavachin	*AGTR1*	0.104672
FBB151	Isobavachin	*ALOX5*	0.104672
FBB151	Isobavachin	*AR*	0.104672
FBB151	Isobavachin	*BRAF*	0.104672
FBB151	Isobavachin	*CA12*	0.104672
FBB151	Isobavachin	*CA5A*	0.104672
FBB151	Isobavachin	*CA5B*	0.104672
FBB151	Isobavachin	*CMA1*	0.104672
FBB151	Isobavachin	*ERBB2*	0.104672
FBB151	Isobavachin	*GSK3B*	0.104672
FBB151	Isobavachin	*LCK*	0.104672
FBB151	Isobavachin	*MDM4*	0.104672
FBB151	Isobavachin	*MET*	0.104672
FBB151	Isobavachin	*MMP12*	0.104672
FBB151	Isobavachin	*MMP15*	0.104672
FBB151	Isobavachin	*MMP16*	0.104672
FBB151	Isobavachin	*MMP3*	0.104672
FBB151	Isobavachin	*MTOR*	0.104672
FBB151	Isobavachin	*NR1H4*	0.104672
FBB151	Isobavachin	*OPRM1*	0.104672
FBB151	Isobavachin	*PLA2G1B*	0.104672
FBB151	Isobavachin	*PLA2G2A*	0.104672
FBB151	Isobavachin	*PLA2G5*	0.104672
FBB151	Isobavachin	*PLA2G7*	0.104672
FBB151	Isobavachin	*PLAU*	0.104672
FBB151	Isobavachin	*PRKCZ*	0.104672
FBB151	Isobavachin	*PTGS1*	0.104672
FBB151	Isobavachin	*PTGS2*	0.104672
FBB151	Isobavachin	*PTPN1*	0.104672

ID	Compounds	gene	Probability
FBB151	Isobavachin	*RXRA*	0.104672
FBB151	Isobavachin	*SERPINE1*	0.104672
FBB151	Isobavachin	*SLC5A2*	0.104672
FBB151	Isobavachin	*SRC*	0.104672
FBB151	Isobavachin	*TRPV1*	0.104672
FBB151	Isobavachin	*TYMS*	0.104672
FBB151	Isobavachin	*WEE1*	0.104672
FBB43	(3S)-5,7-dihydroxy-3-[(4-methoxyphenyl)methyl]-6-methyl-2,3-dihydrochromen-4-one*	*ABL1*	0.101614
FBB43	(3S)-5,7-dihydroxy-3-[(4-methoxyphenyl)methyl]-6-methyl-2,3-dihydrochromen-4-one*	*ADAM10*	0.101614
FBB43	(3S)-5,7-dihydroxy-3-[(4-methoxyphenyl)methyl]-6-methyl-2,3-dihydrochromen-4-one*	*AKT1*	0.101614
FBB43	(3S)-5,7-dihydroxy-3-[(4-methoxyphenyl)methyl]-6-methyl-2,3-dihydrochromen-4-one*	*ALOX5*	0.101614
FBB43	(3S)-5,7-dihydroxy-3-[(4-methoxyphenyl)methyl]-6-methyl-2,3-dihydrochromen-4-one*	*ANPEP*	0.101614
FBB43	(3S)-5,7-dihydroxy-3-[(4-methoxyphenyl)methyl]-6-methyl-2,3-dihydrochromen-4-one*	*CA4*	0.101614
FBB43	(3S)-5,7-dihydroxy-3-[(4-methoxyphenyl)methyl]-6-methyl-2,3-dihydrochromen-4-one*	*CASP3*	0.101614
FBB43	(3S)-5,7-dihydroxy-3-[(4-methoxyphenyl)methyl]-6-methyl-2,3-dihydrochromen-4-one*	*CCND1*	0.101614
FBB43	(3S)-5,7-dihydroxy-3-[(4-methoxyphenyl)methyl]-6-methyl-2,3-dihydrochromen-4-one*	*CCNA1*	0.101614
FBB43	(3S)-5,7-dihydroxy-3-[(4-methoxyphenyl)methyl]-6-methyl-2,3-dihydrochromen-4-one*	*CDK5*	0.101614
FBB43	(3S)-5,7-dihydroxy-3-[(4-methoxyphenyl)methyl]-6-methyl-2,3-dihydrochromen-4-one*	*CDK7*	0.101614
FBB43	(3S)-5,7-dihydroxy-3-[(4-methoxyphenyl)methyl]-6-methyl-2,3-dihydrochromen-4-one*	*CMA1*	0.101614
FBB43	(3S)-5,7-dihydroxy-3-[(4-methoxyphenyl)methyl]-6-methyl-2,3-dihydrochromen-4-one*	*CTSB*	0.101614
FBB43	(3S)-5,7-dihydroxy-3-[(4-methoxyphenyl)methyl]-6-methyl-2,3-dihydrochromen-4-one*	*CTSS*	0.101614
FBB43	(3S)-5,7-dihydroxy-3-[(4-methoxyphenyl)methyl]-6-methyl-2,3-dihydrochromen-4-one*	*EPHB2*	0.101614
FBB43	(3S)-5,7-dihydroxy-3-[(4-methoxyphenyl)methyl]-6-methyl-2,3-dihydrochromen-4-one*	*EZR*	0.101614
FBB43	(3S)-5,7-dihydroxy-3-[(4-methoxyphenyl)methyl]-6-methyl-2,3-dihydrochromen-4-one*	*FGFR1*	0.101614
FBB43	(3S)-5,7-dihydroxy-3-[(4-methoxyphenyl)methyl]-6-methyl-2,3-dihydrochromen-4-one*	*FLT3*	0.101614
FBB43	(3S)-5,7-dihydroxy-3-[(4-methoxyphenyl)methyl]-6-methyl-2,3-dihydrochromen-4-one*	*FOS*	0.101614

ID	Compounds	gene	Probability
FBB43	(3S)-5,7-dihydroxy-3-[(4-methoxyphenyl)methyl]-6-methyl-2,3-dihydrochromen-4-one*	*GRM5*	0.101614
FBB43	(3S)-5,7-dihydroxy-3-[(4-methoxyphenyl)methyl]-6-methyl-2,3-dihydrochromen-4-one*	*GSK3B*	0.101614
FBB43	(3S)-5,7-dihydroxy-3-[(4-methoxyphenyl)methyl]-6-methyl-2,3-dihydrochromen-4-one*	*HDAC1*	0.101614
FBB43	(3S)-5,7-dihydroxy-3-[(4-methoxyphenyl)methyl]-6-methyl-2,3-dihydrochromen-4-one*	*HTT*	0.101614
FBB43	(3S)-5,7-dihydroxy-3-[(4-methoxyphenyl)methyl]-6-methyl-2,3-dihydrochromen-4-one*	*KIT*	0.101614
FBB43	(3S)-5,7-dihydroxy-3-[(4-methoxyphenyl)methyl]-6-methyl-2,3-dihydrochromen-4-one*	*MAPK1*	0.101614
FBB43	(3S)-5,7-dihydroxy-3-[(4-methoxyphenyl)methyl]-6-methyl-2,3-dihydrochromen-4-one*	*MAPKAPK2*	0.101614
FBB43	(3S)-5,7-dihydroxy-3-[(4-methoxyphenyl)methyl]-6-methyl-2,3-dihydrochromen-4-one*	*MMP12*	0.101614
FBB43	(3S)-5,7-dihydroxy-3-[(4-methoxyphenyl)methyl]-6-methyl-2,3-dihydrochromen-4-one*	*MMP16*	0.101614
FBB43	(3S)-5,7-dihydroxy-3-[(4-methoxyphenyl)methyl]-6-methyl-2,3-dihydrochromen-4-one*	*MMP25*	0.101614
FBB43	(3S)-5,7-dihydroxy-3-[(4-methoxyphenyl)methyl]-6-methyl-2,3-dihydrochromen-4-one*	*MTOR*	0.101614
FBB43	(3S)-5,7-dihydroxy-3-[(4-methoxyphenyl)methyl]-6-methyl-2,3-dihydrochromen-4-one*	*PIK3CA*	0.101614
FBB43	(3S)-5,7-dihydroxy-3-[(4-methoxyphenyl)methyl]-6-methyl-2,3-dihydrochromen-4-one*	*PIK3CD*	0.101614
FBB43	(3S)-5,7-dihydroxy-3-[(4-methoxyphenyl)methyl]-6-methyl-2,3-dihydrochromen-4-one*	*PIK3R1*	0.101614
FBB43	(3S)-5,7-dihydroxy-3-[(4-methoxyphenyl)methyl]-6-methyl-2,3-dihydrochromen-4-one*	*PLA2G1B*	0.101614
FBB43	(3S)-5,7-dihydroxy-3-[(4-methoxyphenyl)methyl]-6-methyl-2,3-dihydrochromen-4-one*	*PLA2G7*	0.101614
FBB43	(3S)-5,7-dihydroxy-3-[(4-methoxyphenyl)methyl]-6-methyl-2,3-dihydrochromen-4-one*	*PPARG*	0.101614
FBB43	(3S)-5,7-dihydroxy-3-[(4-methoxyphenyl)methyl]-6-methyl-2,3-dihydrochromen-4-one*	*PTGS2*	0.101614
FBB43	(3S)-5,7-dihydroxy-3-[(4-methoxyphenyl)methyl]-6-methyl-2,3-dihydrochromen-4-one*	*PTPN1*	0.101614
FBB43	(3S)-5,7-dihydroxy-3-[(4-methoxyphenyl)methyl]-6-methyl-2,3-dihydrochromen-4-one*	*ROCK1*	0.101614
FBB43	(3S)-5,7-dihydroxy-3-[(4-methoxyphenyl)methyl]-6-methyl-2,3-dihydrochromen-4-one*	*RPS6KA2*	0.101614
FBB43	(3S)-5,7-dihydroxy-3-[(4-methoxyphenyl)methyl]-6-methyl-2,3-dihydrochromen-4-one*	*SERPINE1*	0.101614

ID	Compounds	gene	Probability
FBB43	(3S)-5,7-dihydroxy-3-[(4-methoxyphenyl)methyl]-6-methyl-2,3-dihydrochromen-4-one*	*SIRT2*	0.101614
FBB43	(3S)-5,7-dihydroxy-3-[(4-methoxyphenyl)methyl]-6-methyl-2,3-dihydrochromen-4-one*	*SRC*	0.101614
FBB43	(3S)-5,7-dihydroxy-3-[(4-methoxyphenyl)methyl]-6-methyl-2,3-dihydrochromen-4-one*	*VCP*	0.101614
FBB43	(3S)-5,7-dihydroxy-3-[(4-methoxyphenyl)methyl]-6-methyl-2,3-dihydrochromen-4-one*	*WEE1*	0.101614
FBB66	Sexangularetin	*ACHE*	0.101614
FBB66	Sexangularetin	*ADORA3*	0.101614
FBB66	Sexangularetin	*AKR1A1*	0.101614
FBB66	Sexangularetin	*AKR1C2*	0.101614
FBB66	Sexangularetin	*AKT1*	0.101614
FBB66	Sexangularetin	*ALK*	0.101614
FBB66	Sexangularetin	*AXL*	0.101614
FBB66	Sexangularetin	*CA5A*	0.101614
FBB66	Sexangularetin	*CDK5*	0.101614
FBB66	Sexangularetin	*CXCR1*	0.101614
FBB66	Sexangularetin	*CYP1A1*	0.101614
FBB66	Sexangularetin	*CYP1A2*	0.101614
FBB66	Sexangularetin	*DAPK1*	0.101614
FBB66	Sexangularetin	*ESR1*	0.101614
FBB66	Sexangularetin	*F2*	0.101614
FBB66	Sexangularetin	*GPR35*	0.101614
FBB66	Sexangularetin	*GRK6*	0.101614
FBB66	Sexangularetin	*GSK3B*	0.101614
FBB66	Sexangularetin	*KDR*	0.101614
FBB66	Sexangularetin	*MAPT*	0.101614
FBB66	Sexangularetin	*MET*	0.101614
FBB66	Sexangularetin	*MMP2*	0.101614
FBB66	Sexangularetin	*MMP3*	0.101614
FBB66	Sexangularetin	*MMP9*	0.101614
FBB66	Sexangularetin	*MPO*	0.101614
FBB66	Sexangularetin	*PIK3R1*	0.101614
FBB66	Sexangularetin	*PLA2G1B*	0.101614
FBB66	Sexangularetin	*PLG*	0.101614
FBB66	Sexangularetin	*PTK2*	0.101614
FBB66	Sexangularetin	*SRC*	0.101614
FBB125	3-O-Acetylpinobanksin	*ABCC1*	0.101614
FBB125	3-O-Acetylpinobanksin	*ADORA1*	0.101614
FBB125	3-O-Acetylpinobanksin	*ADORA3*	0.101614
FBB125	3-O-Acetylpinobanksin	*ALK*	0.101614
FBB125	3-O-Acetylpinobanksin	*BCL2*	0.101614
FBB125	3-O-Acetylpinobanksin	*CA5A*	0.101614
FBB125	3-O-Acetylpinobanksin	*CA5B*	0.101614
FBB125	3-O-Acetylpinobanksin	*CCND1*	0.101614
FBB125	3-O-Acetylpinobanksin	*CDC25A*	0.101614
FBB125	3-O-Acetylpinobanksin	*CCNA1*	0.101614

ID	Compounds	gene	Probability
FBB125	3-O-Acetylpinobanksin	*CDK5*	0.101614
FBB125	3-O-Acetylpinobanksin	*CMA1*	0.101614
FBB125	3-O-Acetylpinobanksin	*CTSG*	0.101614
FBB125	3-O-Acetylpinobanksin	*CXCR1*	0.101614
FBB125	3-O-Acetylpinobanksin	*CXCR2*	0.101614
FBB125	3-O-Acetylpinobanksin	*ESR1*	0.101614
FBB125	3-O-Acetylpinobanksin	*ESR2*	0.101614
FBB125	3-O-Acetylpinobanksin	*FLT3*	0.101614
FBB125	3-O-Acetylpinobanksin	*FUT4*	0.101614
FBB125	3-O-Acetylpinobanksin	*GSK3B*	0.101614
FBB125	3-O-Acetylpinobanksin	*GUSB*	0.101614
FBB125	3-O-Acetylpinobanksin	*HDAC3*	0.101614
FBB125	3-O-Acetylpinobanksin	*HSD11B1*	0.101614
FBB125	3-O-Acetylpinobanksin	*KCNMA1*	0.101614
FBB125	3-O-Acetylpinobanksin	*KDR*	0.101614
FBB125	3-O-Acetylpinobanksin	*MAP2K1*	0.101614
FBB125	3-O-Acetylpinobanksin	*MAPK1*	0.101614
FBB125	3-O-Acetylpinobanksin	*MAPK14*	0.101614
FBB125	3-O-Acetylpinobanksin	*MAPKAPK2*	0.101614
FBB125	3-O-Acetylpinobanksin	*MMP7*	0.101614
FBB125	3-O-Acetylpinobanksin	*MTOR*	0.101614
FBB125	3-O-Acetylpinobanksin	*PIK3CA*	0.101614
FBB125	3-O-Acetylpinobanksin	*PIM1*	0.101614
FBB125	3-O-Acetylpinobanksin	*PLA2G1B*	0.101614
FBB125	3-O-Acetylpinobanksin	*PTGS1*	0.101614
FBB125	3-O-Acetylpinobanksin	*ROCK1*	0.101614
FBB125	3-O-Acetylpinobanksin	*RXRA*	0.101614
FBB125	3-O-Acetylpinobanksin	*SLC9A1*	0.101614
FBB125	3-O-Acetylpinobanksin	*SRC*	0.101614
FBB125	3-O-Acetylpinobanksin	*STAT1*	0.101614
FBB125	3-O-Acetylpinobanksin	*TOP1*	0.101614
FBB47	Glabrescione B	*ABCB1*	0.100634
FBB47	Glabrescione B	*ACHE*	0.100634
FBB47	Glabrescione B	*ADORA1*	0.100634
FBB47	Glabrescione B	*ADORA2A*	0.100634
FBB47	Glabrescione B	*ALDH2*	0.100634
FBB47	Glabrescione B	*BRAF*	0.100634
FBB47	Glabrescione B	*BTK*	0.100634
FBB47	Glabrescione B	*CA12*	0.100634
FBB47	Glabrescione B	*CASP1*	0.100634
FBB47	Glabrescione B	*CASP3*	0.100634
FBB47	Glabrescione B	*CCND1*	0.100634
FBB47	Glabrescione B	*CSF1R*	0.100634
FBB47	Glabrescione B	*CTSK*	0.100634
FBB47	Glabrescione B	*CTSS*	0.100634
FBB47	Glabrescione B	*DPP4*	0.100634
FBB47	Glabrescione B	*EGFR*	0.100634
FBB47	Glabrescione B	*ESR2*	0.100634
FBB47	Glabrescione B	*F10*	0.100634

ID	Compounds	gene	Probability
FBB47	Glabrescione B	*GRM5*	0.100634
FBB47	Glabrescione B	*HDAC1*	0.100634
FBB47	Glabrescione B	*HTR1A*	0.100634
FBB47	Glabrescione B	*IKBKB*	0.100634
FBB47	Glabrescione B	*IL2*	0.100634
FBB47	Glabrescione B	*JAK2*	0.100634
FBB47	Glabrescione B	*LCK*	0.100634
FBB47	Glabrescione B	*MAPK10*	0.100634
FBB47	Glabrescione B	*MAPK14*	0.100634
FBB47	Glabrescione B	*MAPK8*	0.100634
FBB47	Glabrescione B	*MAPK9*	0.100634
FBB47	Glabrescione B	*MIF*	0.100634
FBB47	Glabrescione B	*MTOR*	0.100634
FBB47	Glabrescione B	*NTRK1*	0.100634
FBB47	Glabrescione B	*PDE10A*	0.100634
FBB47	Glabrescione B	*PDE5A*	0.100634
FBB47	Glabrescione B	*PIK3CA*	0.100634
FBB47	Glabrescione B	*PPARA*	0.100634
FBB47	Glabrescione B	*PSEN2*	0.100634
FBB47	Glabrescione B	*PTAFR*	0.100634
FBB47	Glabrescione B	*PTGES*	0.100634
FBB47	Glabrescione B	*PTPRS*	0.100634
FBB47	Glabrescione B	*RPS6KA2*	0.100634
FBB47	Glabrescione B	*SLC2A1*	0.100634
FBB47	Glabrescione B	*SLC6A2*	0.100634
FBB47	Glabrescione B	*SRC*	0.100634
FBB47	Glabrescione B	*TLR9*	0.100634
FBB47	Glabrescione B	*TRPV1*	0.100634
FBB47	Glabrescione B	*WNT3A*	0.100634
FBB38	7-Hydroxyflavone	*AR*	0.098947
FBB38	7-Hydroxyflavone	*CFTR*	0.098947
FBB38	7-Hydroxyflavone	*GSK3B*	0.098947
FBB38	7-Hydroxyflavone	*PTGS2*	0.089872
FBB38	7-Hydroxyflavone	*APP*	0.080792
FBB38	7-Hydroxyflavone	*CD38*	0.080792
FBB38	7-Hydroxyflavone	*MMP12*	0.080792
FBB38	7-Hydroxyflavone	*MMP2*	0.080792
FBB38	7-Hydroxyflavone	*MMP9*	0.080792
FBB38	7-Hydroxyflavone	*TOP1*	0.080792
FBB38	7-Hydroxyflavone	*CYP1A1*	0.071716
FBB38	7-Hydroxyflavone	*MAPK3*	0.071716
FBB38	7-Hydroxyflavone	*PDE5A*	0.071716
FBB38	7-Hydroxyflavone	*PLA2G2A*	0.071716
FBB38	7-Hydroxyflavone	*PLA2G4A*	0.071716
FBB38	7-Hydroxyflavone	*ADORA3*	0.062622
FBB38	7-Hydroxyflavone	*CYP1A2*	0.062622
FBB38	7-Hydroxyflavone	*KIT*	0.062622
FBB38	7-Hydroxyflavone	*PFKFB3*	0.062622
FBB38	7-Hydroxyflavone	*PLG*	0.062622

ID	Compounds	gene	Probability
FBB38	7-Hydroxyflavone	*ST6GAL1*	0.062622
FBB38	7-Hydroxyflavone	*TERT*	0.062622
FBB38	7-Hydroxyflavone	*DAPK1*	0.053556
FBB38	7-Hydroxyflavone	*F2*	0.053556
FBB38	7-Hydroxyflavone	*GPR35*	0.053556
FBB38	7-Hydroxyflavone	*IGF1R*	0.053556
FBB38	7-Hydroxyflavone	*INSR*	0.053556
FBB38	7-Hydroxyflavone	*MAPT*	0.053556
FBB38	7-Hydroxyflavone	*MMP3*	0.053556
FBB38	7-Hydroxyflavone	*MPO*	0.053556
FBB38	7-Hydroxyflavone	*MYLK*	0.053556
FBB38	7-Hydroxyflavone	*PIK3R1*	0.053556
FBB38	7-Hydroxyflavone	*PTK2*	0.053556
FBB38	7-Hydroxyflavone	*TOP2A*	0.053556

Table S3

Core targets of FBB for the treatment of lung injury

Degree	Target gene	Degree	Target gene
38	*TP53*	14	*MAPK14*
33	*SRC*	14	*HIF1A*
33	*PIK3R1*	14	*MAPK9*
32	*STAT3*	14	*IGF1R*
31	*PIK3CA*	13	*TNF*
29	*AKT1*	13	*PTGS2*
28	*MAPK1*	13	*HDAC1*
28	*MAPK3*	13	*ERBB2*
28	*PIK3CD*	12	*MDM2*
25	*ESR1*	12	*GSK3B*
24	*HRAS*	12	*STAT1*
21	*EP300*	12	*KDR*
20	*EGFR*	11	*CASP3*
19	*PTK2*	11	*NCOR2*
18	*JAK2*	11	*PRKCE*
17	*BCL2*	11	*PDGFRA*
16	*MAPK8*	11	*PDGFRB*
15	*CCND1*	10	*ESR2*
15	*FOS*	10	*MAP3K7*

Table S4

PCR primer sequences

Primer name	Primer sequences
GAPDH（F）	AGGTCGGTGTGAACGGATTTG
GAPDH（R）	TGTAGACCATGTAGTTGAGGTCA
iNOS（F）	GAACTGTAGCACAGCACAGGAAAT
iNOS（R）	CGTACCGGATGAGCTGTGAAT
IL-6（F）	CCAGAAACCGCTATGAAGTTCC
IL-6（R）	GTTGGGAGTGGTATCCTCTGTGA
IL-1β（F）	GTTCCCATTAGACAACTGCACTACAG
IL-1β（R）	GTCGTTGCTTGGTTCTCCTTGTA
TNF-α（F）	CCCCAAAGGGATGAGAAGTTC
TNF-α（R）	CCTCCACTTGGTGGTTTGCT
COX-2（F）	TTCAACACACTCTATCACTGGC
COX-2（R）	AGAAGCGTTTGCGGTACTCAT

Table S5

Inflammatory cytokine and receptor-related genes

Gene classification (Mouse)	***Related genes***
Chemokines	*Ccl1*, *Ccl11*, *Ccl12*, *Ccl17*, *Ccl19*, *Ccl2*, *Ccl20*, *Ccl22*, *Ccl24*, *Ccl3*, *Ccl4*, *Ccl5*, *Ccl6*, *Ccl7*, *Ccl8*, *Ccl9*, *Cx3cl1*, *Cxcl1*, *Cxcl10*, *Cxcl11*, *Cxcl12*, *Cxcl13*, *Cxcl15*, *Cxcl5*, *Cxcl9*
Chemokine receptors	*Ccr1*, *Ccr10*, *Ccr2*, *Ccr3*, *Ccr4*, *Ccr5*, *Ccr6*, *Ccr8*, *Cxcr2*, *Cxcr3*, *Cxcr5*
Interleukins	*Il-11*, *Il-13*, *Il-15*, *Il-16*, *Il-17a*, *Il-17b*, *Il-17f*, *Il-1a*, *Il-1b*, *Il-1rn*, *Il-21*, *Il-27*, *Il-3*, *Il-33*, *Il-4*, *Il-5*, *Il-7*
Interleukin receptors	*Il-10ra*, *Il-10rb*, *Il-1r1*, *Il-1r2*, *Il-2rb*, *Il-2rg*, *Il-5ra*, *Il-6ra*, *Il-6st*
Other cytokines	*Aimp1*, *Bmp2*, *Cd40lg*, *Csf1*, *Csf2*, *Csf3*, *Fasl*, *Ifng*, *Lta*, *Ltb*, *Mif*, *Nampt*, *Osm*, *Pf4*, *Spp1*, *Tnf*, *Tnfsf10*, *Tnfsf11*, *Tnfsf13*, *Tnfsf13b*, *Tnfsf4*, *Vegfa*
Other cytokine receptors	*Tnfrsf11b*

NOTE: Please save this file locally before filling in the table, DO NOT work on the file within your internet browser as changes will not be saved. Adobe Acrobat Reader (available free here) is recommended for completion.

The ARRIVE guidelines 2.0: author checklist

The ARRIVE Essential 10

These items are the basic minimum to include in a manuscript. Without this information, readers and reviewers cannot assess the reliability of the findings.

Item		Recommendation	Section/line number, or reason for not reporting
**Study design**	1	For each experiment, provide brief details of study design including:	
		a. The groups being compared, including control groups. If no control group has been used, the rationale should be stated.	P14/L7
		b. The experimental unit (e.g. a single animal, litter, or cage of animals).	P14/L6
**Sample size**	2	a. Specify the exact number of experimental units allocated to each group, and the total number in each experiment. Also indicate the total number of animals used.	P14/L6
		b. Explain how the sample size was decided. Provide details of any *a priori* sample size calculation, if done.	Calculated but not described
**Inclusion and exclusion criteria**	3	a. Describe any criteria used for including and excluding animals (or experimental units) during the experiment, and data points during the analysis. Specify if these criteria were established *a priori*. If no criteria were set, state this explicitly.	P12/L37
		b. For each experimental group, report any animals, experimental units or data points not included in the analysis and explain why. If there were no exclusions, state so.	Mice that died due to health
		c. For each analysis, report the exact value of *n* in each experimental group.	P14/L6;P16/L17
**Randomisation**	4	a. State whether randomisation was used to allocate experimental units to control and treatment groups. If done, provide the method used to generate the randomisation sequence.	P14/L6
		b. Describe the strategy used to minimise potential confounders such as the order of treatments and measurements, or animal/cage location. If confounders were not controlled, state this explicitly.	Randomization is considered sufficient
**Blinding**	5	Describe who was aware of the group allocation at the different stages of the experiment (during the allocation, the conduct of the experiment, the outcome assessment, and the data analysis).	Blinding was not enforced
**Outcome measures**	6	a. Clearly define all outcome measures assessed (e.g. cell death, molecular markers, or behavioural changes).	P14/L12;P14/L17;P14/L23;P14/L30; P14/L39;P15/L6;P15/L17;P15/L29
		b. For hypothesis-testing studies, specify the primary outcome measure, i.e. the outcome measure that was used to determine the sample size.	NA
**Statistical methods**	7	a. Provide details of the statistical methods used for each analysis, including software used.	P16/L11
		b. Describe any methods used to assess whether the data met the assumptions of the statistical approach, and what was done if the assumptions were not met.	P16/L11
**Experimental animals**	8	a. Provide species-appropriate details of the animals used, including species, strain and substrain, sex, age or developmental stage, and, if relevant, weight.	P12/L37
		b. Provide further relevant information on the provenance of animals, health/immune status, genetic modification status, genotype, and any previous procedures.	P12/L38
**Experimental procedures**	9	For each experimental group, including controls, describe the procedures in enough detail to allow others to replicate them, including:	
		a. What was done, how it was done and what was used.	P13/L46
		b. When and how often.	P13/L46
		c. Where (including detail of any acclimatisation periods).	P12/L40
		d. Why (provide rationale for procedures).	P13/L46
**Results**	10	For each experiment conducted, including independent replications, report:	
		a. Summary/descriptive statistics for each experimental group, with a measure of variability where applicable (e.g. mean and SD, or median and range).	P16/L11
		b. If applicable, the effect size with a confidence interval.	P16/L11

The Recommended Set

These items complement the Essential 10 and add important context to the study. Reporting the items in both sets represents best practice.

Item		Recommendation	Section/line number, or reason for not reporting
**Abstract**	11	Provide an accurate summary of the research objectives, animal species, strain and sex, key methods, principal findings, and study conclusions.	P12/L37；P1/L10
**Background**	12	a. Include sufficient scientific background to understand the rationale and context for the study, and explain the experimental approach.	P1/L10
		b. Explain how the animal species and model used address the scientific objectives and, where appropriate, the relevance to human biology.	P14/L7
**Objectives**	13	Clearly describe the research question, research objectives and, where appropriate, specific hypotheses being tested.	P1/L39
**Ethical statement**	14	Provide the name of the ethical review committee or equivalent that has approved the use of animals in this study, and any relevant licence or protocol numbers (if applicable). If ethical approval was not sought or granted, provide a justification.	P12/L44
**Housing and husbandry**	15	Provide details of housing and husbandry conditions, including any environmental enrichment.	P12/L40
**Animal care and monitoring**	16	a. Describe any interventions or steps taken in the experimental protocols to reduce pain, suffering and distress.	P14/L9;P14/L12;P14/L17; P14/L23;P14/L30;P14/L39;
		b. Report any expected or unexpected adverse events.	The event has nothing to do with the experiment
		c. Describe the humane endpoints established for the study, the signs that were monitored and the frequency of monitoring. If the study did not have humane endpoints, state this.	Experimental endpoints occur earlier than distress occurs (eg, acute death under anesthesia)
**Interpretation/ scientific implications**	17	a. Interpret the results, taking into account the study objectives and hypotheses, current theory and other relevant studies in the literature.	P16/L18
		b. Comment on the study limitations including potential sources of bias, limitations of the animal model, and imprecision associated with the results.	P16/L27
**Generalisability/ translation**	18	Comment on whether, and how, the findings of this study are likely to generalise to other species or experimental conditions, including any relevance to human biology (where appropriate).	P16/L34
**Protocol registration**	19	Provide a statement indicating whether a protocol (including the research question, key design features, and analysis plan) was prepared before the study, and if and where this protocol was registered.	P12/L41
**Data access**	20	Provide a statement describing if and where study data are available.	No description of where the data is stored or ho
**Declaration of interests**	21	a. Declare any potential conflicts of interest, including financial and non-financial. If none exist, this should be stated.	No conflicts of interest
		b. List all funding sources (including grant identifier) and the role of the funder(s) in the design, analysis and reporting of the study.	P16/L40
